# GDF15 promotes prostate cancer bone metastasis and colonization through osteoblastic CCL2 and RANKL activation

**DOI:** 10.1038/s41413-021-00178-6

**Published:** 2022-01-20

**Authors:** Jawed Akhtar Siddiqui, Parthasarathy Seshacharyulu, Sakthivel Muniyan, Ramesh Pothuraju, Parvez Khan, Raghupathy Vengoji, Sanjib Chaudhary, Shailendra Kumar Maurya, Subodh Mukund Lele, Maneesh Jain, Kaustubh Datta, Mohd Wasim Nasser, Surinder Kumar Batra

**Affiliations:** 1 Department of Biochemistry and Molecular Biology, University of Nebraska Medical Center, Omaha, NE USA; 2 Department of Pathology and Microbiology, University of Nebraska Medical Center, Omaha, NE USA; 3 Fred & Pamela Buffett Cancer Center, University of Nebraska Medical Center, Omaha, NE 68198-5870 USA; 4 Eppley Institute for Research in Cancer and Allied Diseases, University of Nebraska Medical Center, Omaha, NE USA

**Keywords:** Bone cancer, Bone cancer

## Abstract

Bone metastases occur in patients with advanced-stage prostate cancer (PCa). The cell-cell interaction between PCa and the bone microenvironment forms a vicious cycle that modulates the bone microenvironment, increases bone deformities, and drives tumor growth in the bone. However, the molecular mechanisms of PCa-mediated modulation of the bone microenvironment are complex and remain poorly defined. Here, we evaluated growth differentiation factor-15 (GDF15) function using in vivo preclinical PCa-bone metastasis mouse models and an in vitro bone cell coculture system. Our results suggest that PCa-secreted GDF15 promotes bone metastases and induces bone microarchitectural alterations in a preclinical xenograft model. Mechanistic studies revealed that GDF15 increases osteoblast function and facilitates the growth of PCa in bone by activating osteoclastogenesis through osteoblastic production of CCL2 and RANKL and recruitment of osteomacs. Altogether, our findings demonstrate the critical role of GDF15 in the modulation of the bone microenvironment and subsequent development of PCa bone metastasis.

## Introduction

Prostate cancer (PCa), the most common malignant tumor in men, is the second leading cause of cancer deaths in males worldwide^1^. Patients with advanced PCa frequently exhibit distant metastasis, with bone being the preferential site in nearly 90% of metastatic PCa patients^2^. Skeletal metastasis is one of the leading causes of mortality in PCa patients^3^. Although the 5-year survival rate of advanced PCa patients without bone metastasis is nearly 60%, it substantially decreases to 3% in the presence of bone metastases^3,4^. PCa bone metastasis is accompanied by skeletal-associated complications such as hypercalcemia, intense pain in the bone, bone fractures, and spinal cord compression^5^. The treatment options for PCa-bone metastasis mostly focus on tumor growth and management of pain; hence, the most advanced disease remains incurable.

Bone is an extremely a dynamic organ that undergoes continuous remodeling^6^. The bone microenvironment comprises osteoclasts and osteoblasts that function to resorb and mineralize (form new) bone, respectively. The activities of these two cell populations are tightly coupled to balance bone turnover. Osteoblasts and osteoclasts originate from mesenchymal and hematopoietic stem cells, respectively^6^. The bone microenvironment regularly secretes factors for bone maintenance that inadvertently provide a fertile environment for the establishment and growth of tumors within the bone, leading to a vicious cycle^7^. On the basis of radiological appearance, bone metastatic lesions can be classified as osteolytic, osteoblastic, or mixed. Bone metastases in PCa are osteoblastic in nature, characterized by the net formation of new bone of poor mechanical quality but featuring high levels of markers of bone resorption/osteoclast activity^8^.

Transforming growth factor-β (TGF-β) is well known to support PCa bone metastases^9,10^. Growth differentiation factor-15 (GDF15), also known as MIC-1, is a member of the TGF-β/bone morphogenic protein superfamily. Physiologically, GDF15 has a major role in prenatal development; the regulation of growth, cartilage and bone formation; and cellular responses to inflammation and stress^11^. In adults, GDF15 facilitates tissue repair after acute injuries. GDF15, which is overexpressed in various malignancies, including PCa, is a potential serum marker in different types of cancers and contributes to tumor development and metastasis^11–16^. High serum GDF15 levels have been observed in patients with bone metastasis^17^. Glial-derived neurotrophic factor receptor alpha-like (GFRAL) was recently identified as a receptor for GDF15^18–21^. GFRAL binds to the coreceptor RET (a tyrosine-protein kinase) after binding with GDF15^18–21^. GFRAL expression seems to be primarily confined to the central nervous system and mediates the anorectic effects of GDF15^22^. However, the presence and role of this GDF15/GFRAL/RET axis in PCa progression and bone metastasis remain obscure. Here, we provide preclinical and clinical evidence to establish the role of PCa-secreted GDF15 in bone metastasis and reveal the mechanism underlying GDF15-mediated osteomac recruitment and alteration of the bone microenvironment.

## Results

### GDF15 promotes PCa bone metastasis

**Fig. 1:**
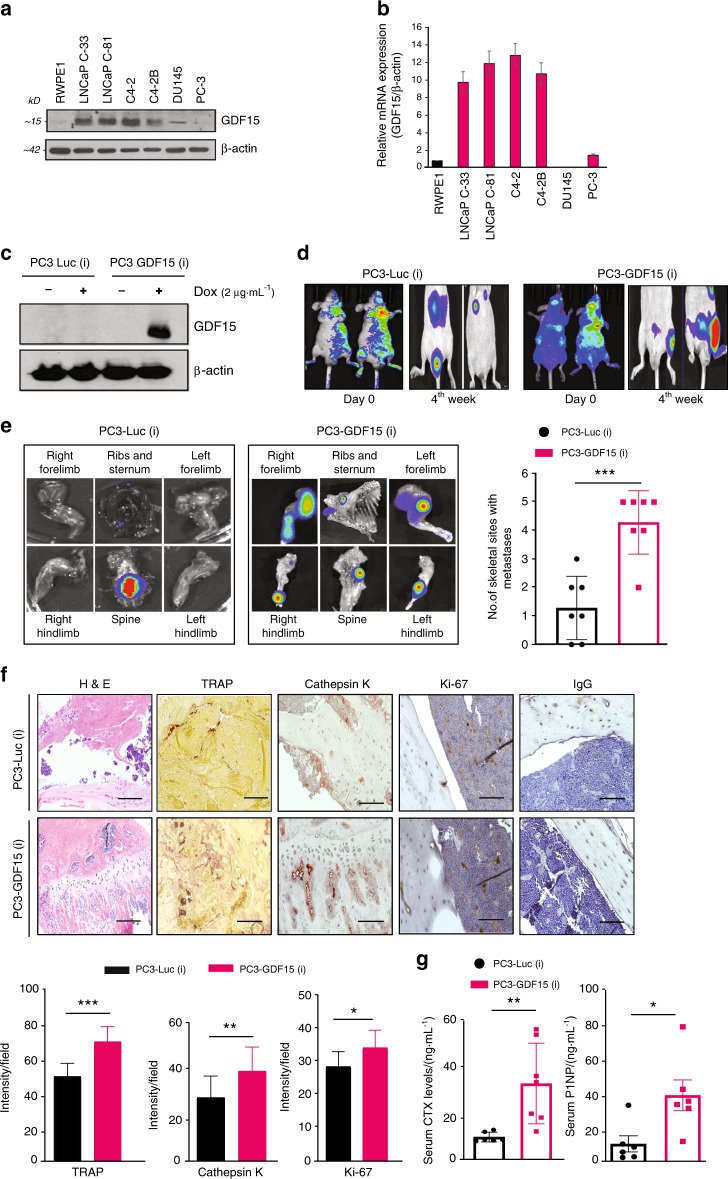
GDF15 promotes PCa bone metastasis. **a** Expression of GDF15 protein in human PCa cell lines as indicated by Western blot analysis. **b** The expression of GDF15 at the transcriptional level was analyzed by RT–qPCR analysis in human PCa cell lines. **c** Western blot analyses to determine GDF15 expression in the lysates of PC3-Luc(i) and PC3-GDF15(i) cells after 48 h of doxycycline (Dox) induction. **d** Representative photographs of mice injected with PC3-Luc(i) and PC3-GDF15(i) cells to detect bone metastasis after Dox treatment at the indicated time points using IVIS imaging. **e** Representative ex vivo images of excised tissues from different skeletal sites after final in vivo imaging within 10 min of luciferin injection (bioluminescent images of the ribs and sternum, spine, forelimbs, and hind limbs) *(left panel)* and quantification of the number of skeletal sites with metastasis in mice injected with PC3-Luc(i) and PC3-GDF15(i) *(right panel)*. **f** Histopathological staining of excised bone sections of PC3-Luc(i)- and PC3-GDF15(i)-injected mice. *(Upper panel)* Representative H&E staining and TRAP (osteoclast marker) staining of bone. Bone sections were immunostained for cathepsin K (a functional marker for bone resorption) and Ki67 and subjected to corresponding IgG staining. *(Lower panel)* Quantification of TRAP, cathepsin K and Ki67 staining. Bone marker analysis in serum obtained from PC3-Luc(i)- and PC3-GDF15-injected mice. Scale bar = 400 µm. **g** Serum CTX and P1NP were measured to assess bone turnover (*n* = 6 mice/group). The data are given as the mean ± SEM, and *P* ≤ 0.05 was considered to indicate statistical significance. *P < 0.05, **P < 0.01, ***P < 0.001 compared to the PC3-Luc(i)-injected group

To determine the clinical significance of GDF15 expression, six Oncomine datasets were evaluated. These datasets showed that GDF15 is overexpressed in PCa patients compared to healthy subjects (Fig. S1a). We further analyzed the TCGA database for GDF15 expression and observed significant upregulation of GDF15 in PCa patients and further increased GDF15 expression in metastatic PCa patients (Fig. S1b and S1c). Next, we examined GDF15 expression in a PCa tissue microarray (TMA), which showed higher staining of GDF15 in prostate adenocarcinoma than in normal prostate samples and increased expression with disease progression (Fig. S1d). We found similar increases in the expression of GDF15 at both the protein and mRNA levels in a panel of human PCa cells compared to normal immortalized RWPE1 cells (Fig. 1a and b).

Previously, we showed that overexpression of GDF15 enhances the proliferation, migration, and anchorage-independent growth of PCa cells^23^. The PC3 cell line, which has low GDF15 expression, was used as a model to evaluate the functional role of GDF15 in PCa bone metastasis. PC3 cell lines stably expressing doxycycline-inducible GDF15-luciferase or control vector-luciferase (PC3-GDF15-Luc[i] or PC3-Luc[i]) were generated (Fig. 1c) and injected intracardially into male nude mice. Metastatic lesions were visualized via IVIS imaging (Fig. 1d). As shown by ex vivo bioluminescent imaging, PCa cells overexpressing GDF15 exhibited widespread skeletal metastasis (in the hind limbs, forelimbs, spinal cord, and ribs with sternum) after one month of inoculation compared to that of control PC3-Luc(i) cells (Fig. 1e). Mice inoculated with PC3-GDF15(i) cells showed declines in their body weight, while those injected with PC3-Luc(i) cells did not (Fig. S1e). After decalcification, the excised tibiae were processed and examined for histopathological changes and metastatic lesions. H&E staining of tibial sections showed tumor-induced cortical bone deterioration in PC3-GDF15(i)-injected mice (Fig. 1f). Next, we stained the bone sections with tartrate-resistant acid phosphatase (TRAP) to determine the bone resorption capacity of mice with PCa overexpressing GDF15. PC3-GDF15(i)-injected mice exhibited more TRAP-positive cells (osteoclasts) and staining with cathepsin K (a marker of functional osteoclasts) than PC3-Luc(i)-implanted mice (Fig. 1f). PC3-GDF15(i)-injected mice also showed more Ki67 staining than PC3-Luc(i) mice (Fig. 1f). To examine the involvement of GDF15 in PCa-mediated bone turnover, we measured serum P1NP and CTX levels, which reflect the degree of bone formation and bone resorption, respectively. PC3-GDF15(i)-injected mice exhibited significantly higher serum CTX and P1NP than PC3-Luc(i)-injected mice (Fig. 1g). Together, these findings strongly suggest that GDF15 overexpression in PCa cells promotes skeletal metastasis.

### PCa-GDF15 modulates the bone microenvironment and favors tumor growth in bone

**Fig. 2:**
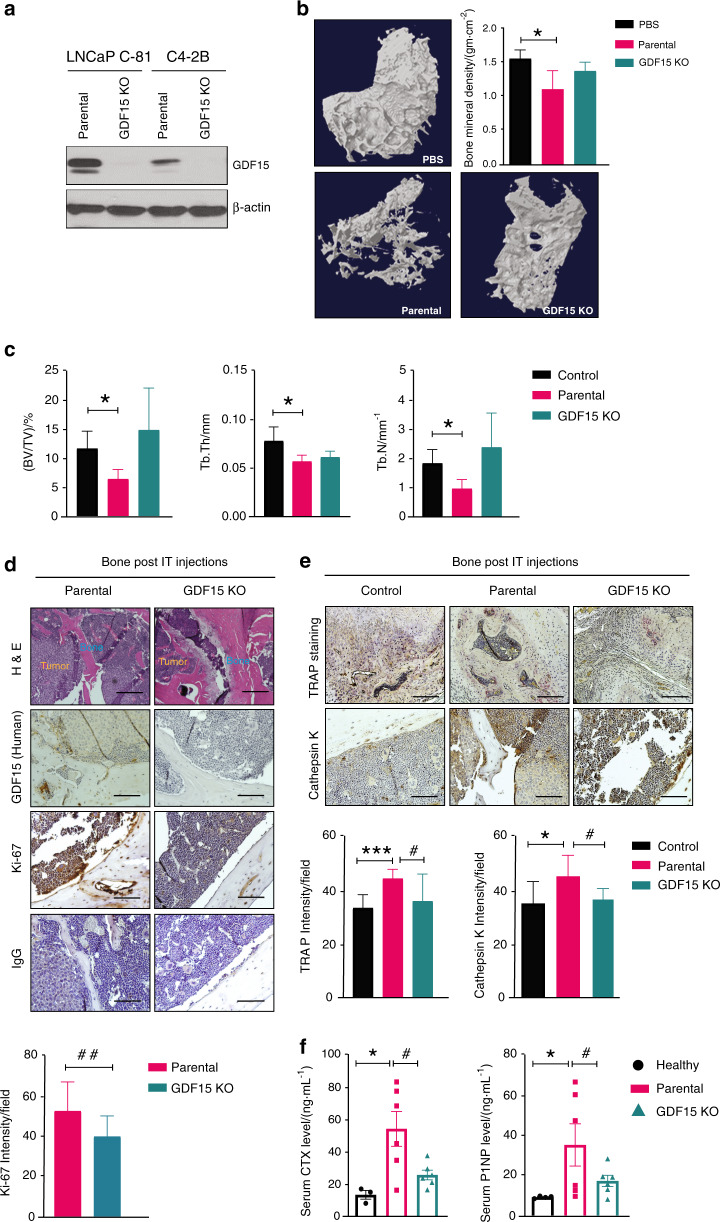
PCa-GDF15 modulates the bone microenvironment and favors tumor growth in bone. **a** Immunoblotting of GDF15 and GDF15 KO clones of the indicated PCa cell lines. **b** Representative 3D reconstruction of µCT images and bone mineral density BMD/(gm·cm^−2^) of the trabecular region of the tibia at 12 weeks after PBS (control), C4-2B (parental cell) or C4-2B (GDF15 KO) injection in mice. **c** µCT analysis of trabecular bone in the tibia showing the bone volume fraction (BV/TV)/%, trabecular thickness (Tb.Th/mm), and trabecular number (Tb.N/mm^−1^). All values are expressed as the mean ± SEM. **d** H&E staining of the excised tibia after decalcification and immunohistochemical staining of tibiae for GDF15 (human), Ki67 and IgG. **e** TRAP and immunohistochemical (cathepsin K) staining of tibiae. Scale bar = 200 µm. **f** Serum assessment of CTX and P1NP. The data are given as the mean ± SEM (*n* = at least 3 mice/group), and *P* ≤ 0.05 was considered to indicate statistical significance. *P < 0.05, ***P < 0.001 compared with the control group and ^#^*P* < 0.05, ^##^*P* < 0.01 compared with the parental (C4-2B)-injected group

To define the role of GDF15 in PCa-mediated modulation of the bone microenvironment, we next generated PCa cell lines (LNCaP C-81 and C4-2B) with GDF15 knockout (KO) using the CRISPR–Cas9 system. Western blot analysis confirmed the complete knockout of GDF15 in LNCaP C-81 and C4-2B cell lines (Fig. 2a). C4-2B (parental and GDF15 KO) PCa cells were inoculated into the tibiae of nude mice. Higher bone destruction was seen in C4-2B-injected tibiae than in PBS control-injected tibiae; however, the deletion of GDF15 prevented bone destruction from PCa, as shown by 3D reconstruction of images obtained from the high-resolution µ-CT analysis of trabecular bone (Fig. 2b). Next, we examined alterations in bone mineral density (BMD) in the tibiae of animals injected with PBS, C4-2B, and C4-2B (GDF15 KO) cells. The BMD was significantly lower in C4-2B cell-injected tibiae than in contralateral nontumor control tibiae; however, deletion of GDF15 did not decrease BMD (Fig. 2b). C4-2B-injected mice showed visible destruction of trabecular bone, while tibiae obtained from the C4-2B (GDF15 KO)-injected mice were comparable to those of PBS-injected control mice (Fig. S2a, Video 1 & Video 2).

We quantified PCa-mediated trabecular deterioration of the tibial epiphyses (sites of the intratibial injections) of C4-2B and C4-2B (GDF15 KO) cell-injected mice at the architectural level by µ-CT analysis. C4-2B-injected mice had significant microarchitectural deterioration, as indicated by reduced bone trabecular volume (BV/TV), trabecular thickness (Tb.Th), connection density (reflecting trabecular connectedness), and trabecular number (Tb.N), and increased trabecular separation (Tb.S). Deletion of GDF15 in C4-2B cells improved bone microarchitectural parameters, such as BV/TV, Tb.Th, connection density, Tb.N and Tb.S, compared to the parameters in GDF15-deficient mice (Figs. 2c and S2b). Furthermore, IHC analysis of the decalcified tibial sections revealed that tumors formed from C4-2B (GDF15 KO) cells had a lower proliferative index than tumors formed from C4-2B cells, as indicated by fewer Ki67‐positive cells (Fig. 2d). The deletion of GDF15 in C4-2B cells also decreased osteoclast formation and function, as reflected by reduced TRAP and cathepsin-K staining (Fig. 2e). Serum analysis of bone turnover markers demonstrated that CTX and P1NP levels were significantly increased in the C4-2B-injected mice compared to the age-matched healthy control mice. In contrast, C4-2B (GDF15 KO)-injected mice showed significantly lower serum CTX and P1NP levels than the C4-2B-injected group (Fig. 2f). Furthermore, we examined the expression of mouse-specific GDF15 in the tibiae of C4-2B- and C4-2B (GDF15 KO)-injected mice. Interestingly, no significant changes were observed in mouse GDF15 expression in the bone of C4-2B PCa-injected mice compared to that of PBS-injected or GDF15 KO cell-injected mice (Fig. S2c). Taken together, these findings indicate that silencing GDF15 impairs tumor growth and progression while protecting the bone microenvironment.

### GDF15 increases the recruitment of bone macrophages (osteomacs) via CCL2

**Fig. 3:**
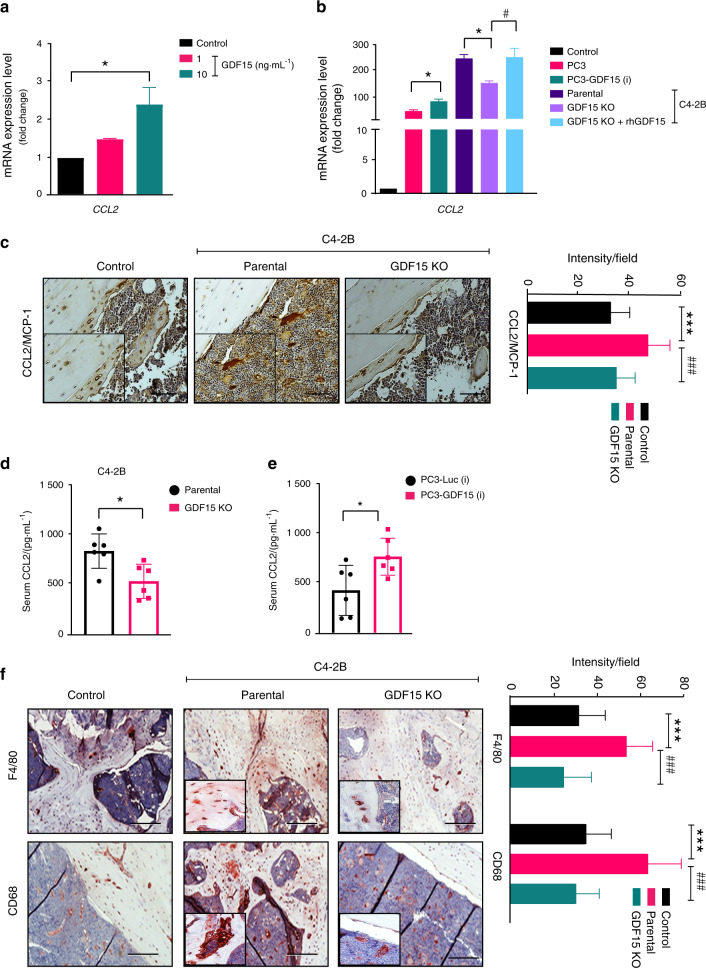
GDF15 increases the recruitment of bone macrophages (osteomacs) via CCL2. GDF15 increases the expression of CCL2 in osteoblasts. **a** Total RNA was isolated from MCOs treated with rhGDF15 for 48 h and was assessed for CCL2 by RT–qPCR. **b** mRNA expression of CCL2 in MCOs after treatment (48 h) with CM from PC3, PC3 cells overexpressing GDF15, C4-2B cells, and C4-2B (GDF15 KO) cells with and without rhGDF15 in vitro. β-Actin was used to normalize the gene expression. **c** Immunohistochemical analysis of CCL2/MCP-1 in tibial sections from C4-2B- and C4-2B (GDF15 KO)-injected mice (*right panel*) quantification. Scale bar = 200 µm. **d** CCL2 levels in serum obtained from C4-2B- and C4-2B (GDF15 KO)-injected mice (*n* = 6 mice/group). **e** Serum CCL2 levels in PC3-Luc(i)- and PC3-GDF15(i)-injected mice (*n* = 6 mice/group). **f** Immunohistochemical analysis of CD68 on F4/80^+^ macrophages in bone sections obtained from C4-2B- and C4-2B (GDF15 KO)-injected tibiae; (*right panel*) quantification (*n* = 3 mice/group). Scale bar = 200 µm. The data are presented as the mean ± SEM, and *P* ≤ 0.05 was considered to indicate statistical significance. *P < 0.05, ***P < 0.001 compared with the control group and ^#^*P* < 0.05, ^###^*P* < 0.001 compared with the parental (C4-2B)-injected group

Bone-resident macrophages, also known as osteomacs, play roles in coordinating normal bone homeostasis and injury healing^24^. Osteomacs have been shown to impact bone metastasis and PCa progression in bone via osteoclast and osteoblast function^25^. Chemokines, particularly C-C family members, affect the development and progression of PCa within the bone microenvironment by modulating the function of bone cells and facilitating the recruitment of macrophages^26,27^. To determine whether GDF15 regulates the expression of C-C chemokines in bone cells, we assessed the mRNA expression of C-C chemokines (CCL2, CCL3, CCL7, and CCL12) in osteoblasts following treatment with either rhGDF15 or conditioned medium (CM) collected from different PCa cells. We found that CCL2 was highly expressed among the analyzed chemokines in osteoblasts treated with rhGDF15 (Figs. 3a and S3a-S3c); CCL2 is well known for macrophage recruitment and PCa growth in the bone microenvironment^26–29^. Similarly, CM from PC3 cells overexpressing GDF15 significantly enhanced CCL2 expression in osteoblasts (Fig. 3b). In contrast, the increase in CCL2 levels was smaller upon treatment with CM from C4-2B (GDF15 KO) cells than upon treatment with CM from C4-2B parental cells. However, C4-2B (GDF15 KO) cell-derived CM supplemented with rhGDF15 induced CCL2 expression to levels comparable to those of parental cells (Fig. 3b). Similar results were observed with CM of the LNCaP C-81 cell line (Fig. S3d). Immunohistochemistry showed significantly higher CCL2 staining in C4-2B-injected tibiae than in either PBS-injected control or C4-2B (GDF15 KO)-injected tibiae (Fig. 3c). In addition, lower serum CCL2 levels were observed in mice injected with GDF15 KO PCa cells than in mice injected with parental PCa cells (Fig. 3d). Mice inoculated with PC3-GDF15(i) cells showed higher serum CCL2 levels than PC3-Luc(i)-injected mice (Fig. 3e). These data suggest that PCa-secreted GDF15 profoundly increases CCL2 expression in osteoblasts.

CCL2 plays a role in macrophage recruitment and fusion^30^. A high degree of infiltration of F4/80^−^ and CD68-positive macrophages was observed in C4-2B-injected tibiae compared to contralateral control tibiae. However, the infiltration of F4/80^−^ and CD68-positive macrophages in C4-2B (GDF15 KO)-injected tibiae was lower than that in tibiae implanted with parental cells and control tibiae (Fig. 3f). Together, these findings suggest that PCa-secreted GDF15 regulates the expression of CCL2, which in turn serves as a chemoattractant that guides osteomacs to the bone surface.

### PCa-secreted GDF15 augments mouse calvarial osteoblast (MCO) activity

**Fig. 4:**
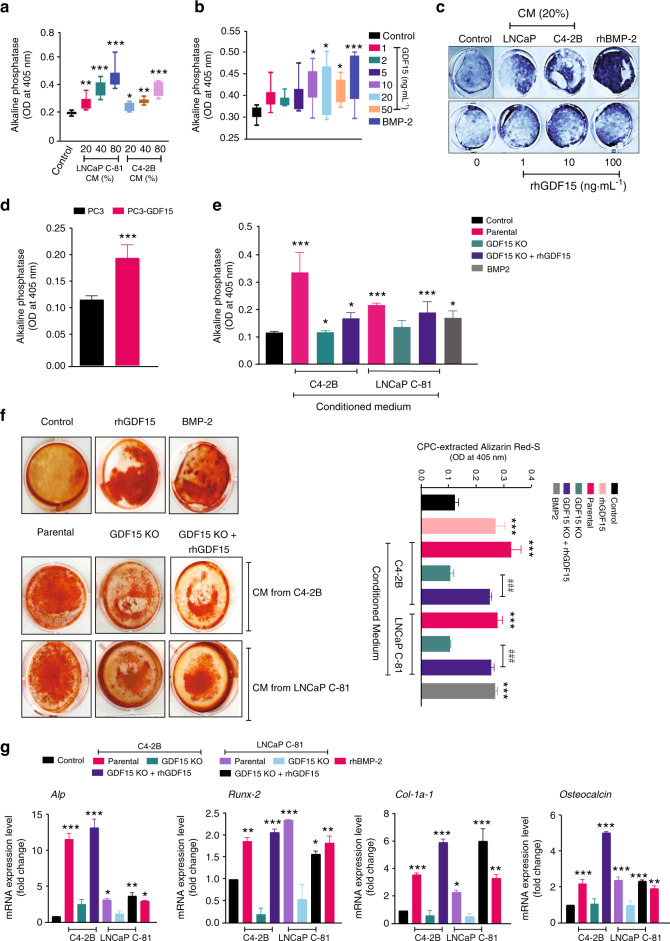
PCa-secreted GDF15 augments osteogenic activity. **a** PCa factors promote osteoblast differentiation, as measured by alkaline phosphatase assay. MCOs were treated with different proportions of conditioned medium (CM 20%, 40%, and 80%) from the LNCaP C-81 and C4-2B cell lines (after 48 h of treatment) and subjected to alkaline phosphatase activity analysis. **b** Treatment with rhGDF15 for 48 h increased osteoblast differentiation, as determined by measuring alkaline phosphatase activity. **c** Representative alkaline phosphatase staining of MCOs after 10 days of treatment with the CM of LNCaP C-81 and C4-2B cells and different doses of rhGDF15. **d, e** Osteoblast differentiation (alkaline phosphatase activity) after 48 h of treatment with **d** CM obtained from PC3 and PC3 cells overexpressing GDF15 and **e** CM from C4-2B and LNCaP C-81 cells. **f** Assessment of mineralized nodules using alizarin red-S dye staining. MCOs were treated with the control, 20% CM from PCa cell lines, and/or rhGDF15 for 21 days, and the osteoblast differentiation medium was replaced every third day. The left panel shows representative alizarin red-S-stained nodules, and the right panel shows quantification of alizarin red-S staining with cetylpyridinium chloride (CPC) extraction. **g** MCOs were treated with the control or 20% CM from PCa cell lines and/or rhGDF15 for 72 h in osteoblast differentiation medium. Total RNA was harvested, RT–qPCR for osteogenic genes (*alkaline phosphatase: ALP, Runx-2, Col-1a, and osteocalcin)* was performed, and gene expression was normalized to that of β-actin. The data represent the mean ± SEM from three independent experiments. *P* ≤ 0.05 was considered to indicate statistical significance

The communication between cancer cells and the bone microenvironment leads to a vicious cycle that induces bone deformities and favors tumor growth in bone. Osteoblasts are the primary bone-forming cells, and PCa patients show high osteoblastic activity. Therefore, we investigated the effect of PCa-secreted GDF15 on MCO differentiation. MCOs were cultured in medium supplemented with increasing concentrations of rhGDF15 or varying proportions of CM from PCa cells (20%, 40%, and 80% in αMEM) under osteoblast-differentiating conditions (in the presence of ascorbic acid and β-glycerophosphate) for 48 h. Treatment with increasing concentrations of CM from cells with high GDF15 (LNCaP C-81 & C4-2B) proportionally increased MCO differentiation after 48 h, as assessed by alkaline phosphatase (Fig. 4a). rhGDF15 also augmented MCO differentiation with increasing concentrations (Fig. 4b and c). Similarly, CM from PC3 cells (which express low endogenous levels of GDF15) engineered to overexpress GDF15 induced greater MCO differentiation than CM from parental cells (Fig. 4d). Next, CM obtained from PCa cells (LNCaP C-81 & C4-2B) with GDF15 deletion inhibited PCa-mediated osteoblast differentiation, and supplementation with rhGDF15 restored osteoblast differentiation (Fig. 4e). These results suggest that GDF15 is crucial for PCa cells to stimulate osteoblast differentiation. We also determined the effect of PCa-secreted GDF15 on osteoblast mineralization, as differentiated MCOs are capable of mineralizing bone matrix. MCO cultures treated with rhGDF15 or CM from LNCaP C-81 and C4-2B cells exhibited robust mineralization, as evidenced by alizarin red-S-stained calcified nodules, and this effect was abrogated upon GDF15 knockdown (Fig. 4f). Gene expression analysis revealed increased mRNA levels of the early osteogenic genes *alkaline phosphatase* and *Runx2* and *the* late osteogenic genes *Col1a1 and osteocalcin* in osteoblasts treated with the CM of PCa cells compared to the control (Fig. 4g). The increased expression of PCa-mediated osteogenic genes was abrogated by GDF15 KO and was partially restored by exogenous supplementation with rhGDF15 (Fig. 4g), suggesting that some aspects of the stimulation of osteoblast differentiation by PCa require GDF15.

Colony-forming unit fibroblasts (CFU-Fs) are recognized as early osteoblastic cell precursors, and the CFU-F assay is an appropriate way to enumerate BMSCs in the bone marrow. To assess the effect of GDF15 on the differentiation of BMSCs into osteoblasts, mouse-derived BMSCs were cultured in bone marrow differentiation medium for 10 and 21 days with and without rhGDF15 or CM from PCa cells. rhGDF15 enhanced the differentiation of BMSCs into osteoblasts and promoted mineralization, as assessed by alkaline phosphatase and alizarin red staining (Fig. S4a and S4b). The CM from GDF15-null C4-2B and LNCaP C-81 cells led to a decrease in BMSC differentiation compared to that of their parental cells, but this effect was reversed by supplementation with rhGDF15 (Fig. S4c). Similarly, PCa-secreted GDF15 increased the mineralization of BMSCs, as indicated by alizarin red-S staining (Fig. S4d). Together, these data suggest that GDF15 contributes to the ability of PCa cells to modulate osteoblastogenic functions, such as differentiation and mineralization.

### PCa-secreted GDF15 induces osteoclastogenesis

**Fig. 5:**
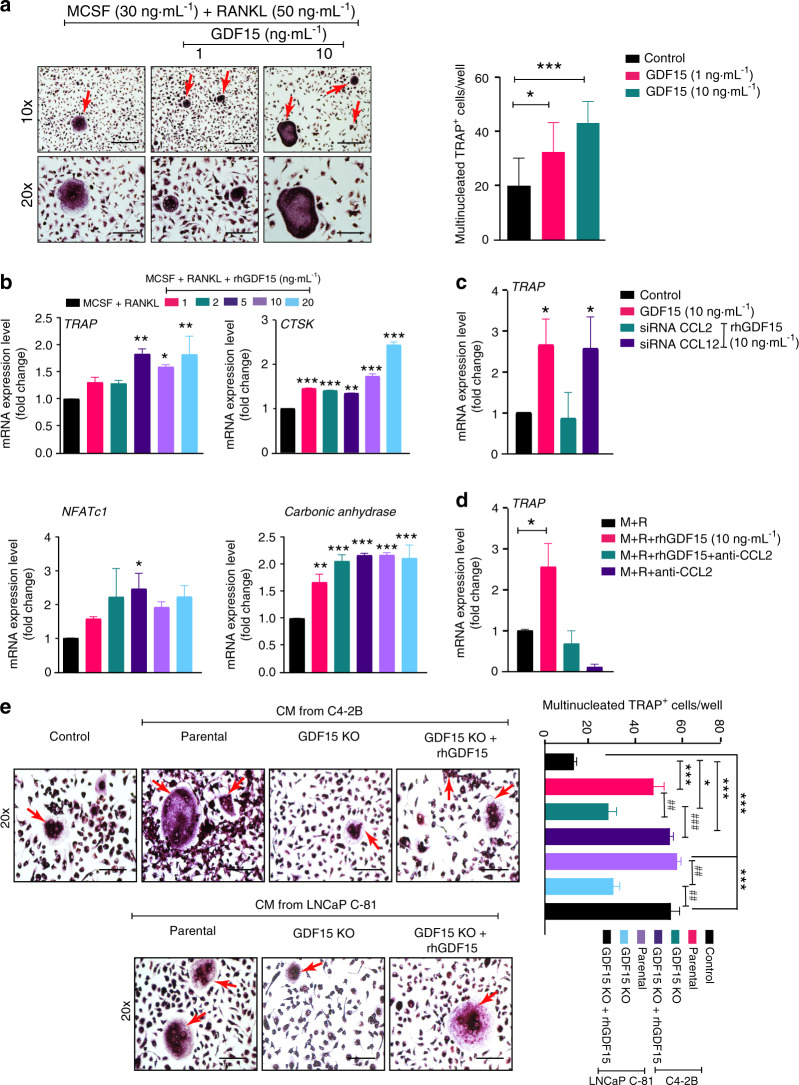
PCa-secreted GDF15 induces osteoclastogenesis. **a** BMMs from C57BL/6J mice were induced to differentiate toward osteoclasts with M-CSF and RANKL in the presence or absence of different concentrations of exogenous rhGDF15 (1 and 10 ng·mL^−1^). After 7 days of culture, the cells were stained for TRAP and photographed by light microscopy to observe the multinucleated TRAP-positive cells (fused cells with 3+ nuclei) known as osteoclasts *(left panel)*. Scale bars = 400 µm (upper) and 200 µm (lower). Quantification of multinucleated TRAP^+^ cells in different experimental groups (*right panel*). **b** To determine the effect of GDF15 on osteoclastogenesis, RT–qPCR analyses showing the mRNA expression of osteoclast markers (*TRAP, cathepsin K, NFATc1, and carbonic anhydrase*) using the same treatment regimen as in (Fig. 5a) were performed. BMMs from C57BL/6J mice were cultured for 7 days in the presence of RANKL and M-CSF and/or rhGDF15 along with **c** siRNA of CCL2 and CCL12 **d** and an anti-CCL2 antibody (10 µg·mL^-1^), and the expression levels of *TRAP* transcripts were analyzed. **e** BMMs cultured in the presence of RANKL and M-CSF and/or rhGDF15 along with CM from C4-2B and LNCaP C-81 cells (parental and GDF15 KO cells). After fixation, the cells were stained for TRAP and photographed by a light microscope *(left panel)*. Scale bar =200 µm, and the results are quantified in the *right panel*. The data represent the mean ± SEM. *P* ≤ 0.05 was considered to indicate statistical significance

Since GDF15 deficiency in PCa cells dramatically reduced TRAP staining (indicating the osteoclast number) in the bone microenvironment, we examined whether GDF15 released by PCa cells might control osteoclast (OC) formation. We used bone marrow from 4-week-old C57BL/6 J mice to generate OCs. Bone marrow macrophages **(**BMMs) from mice were cultured in the presence of M-CSF and RANKL with and without GDF15 for 7 days to stimulate in vitro OC formation. The addition of rhGDF15 resulted in a significant increase in the number of TRAP-positive multinucleated cells (Fig. 5a). Furthermore, we performed RT–qPCR analysis for genes associated with the osteoclastogenesis of BMMs. Osteoclast-specific genes such as *TRAP, cathepsin K, NFATc1*, and *carbonic anhydrase* were elevated after 7 days of rhGDF15 treatment in the presence of M-CSF and RANKL (Fig. 5b). Since our data suggest that PCa-secreted GDF15 increases C-C chemokine (especially CCL2 and CCL12) expression in osteoblasts, we next sought to examine the effect of CCL2 and CCL12 silencing on GDF15-mediated osteoclastogenesis. CCL12 inhibition had no effect, while CCL2 silencing significantly inhibited the GDF15-mediated increase in *TRAP* mRNA expression in cultured BMMs (Figs. 5c and S5a and b). Similarly, treatment of BMMs with a mouse-specific anti-CCL2 antibody inhibited the GDF15-mediated upregulation of *TRAP* expression (Fig. 5d). Similar osteoclastogenic effects were observed following the treatment of BMMs with CM from C4-2B and LNCaP C-81 cells. These effects were abrogated following the deletion of GFD15 and restored upon supplementation of CM with rhGDF15 (Fig. 5e). Together, these data suggest that PCa-secreted GDF15 enhances RANKL-mediated osteoclast differentiation and promotes bone resorption.

### GDF15 increases the osteoclastogenic potential of osteoblasts via an increased RANKL signal

**Fig. 6:**
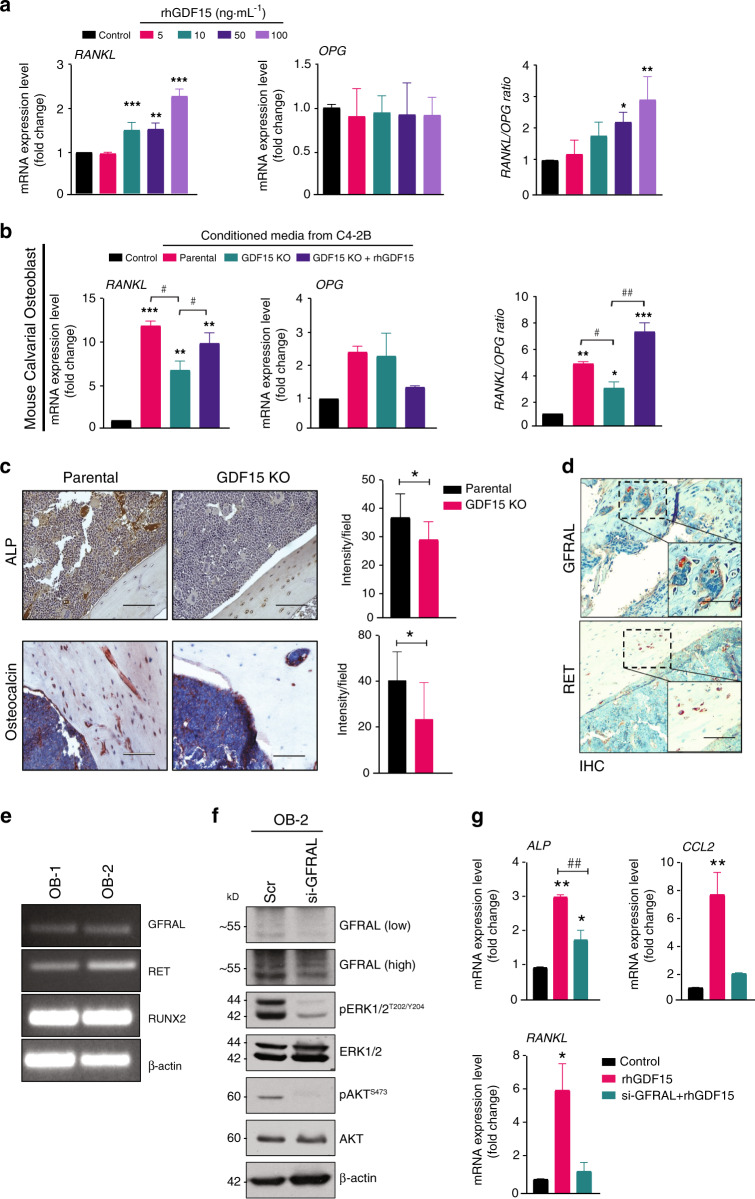
GDF15 increases the osteoclastogenic potential of osteoblastic cells via an increased RANKL signal. **a** MCOs were treated with rhGDF15 for 48 h, and the mRNA expression levels of RANKL and OPG and the ratio of RANKL/OPG expression, representing the degree of osteoclastogenesis, were assessed. **b** RT–qPCR analyses showing the mRNA expression and ratio of RANKL and OPG in MCOs after treatment with CM from C4-2B cell lines (parental and GDF15 KO). **c** Immunohistochemistry of ALP and osteocalcin in bone sections obtained from C4-2B- and C4-2B (GDF15 KO)-injected tibiae; (right panel) quantification (*n* = 3 mice/group). Scale bar = 200 µm. **d** Immunohistochemistry of GFRAL and RET on the decalcified tibiae of PC3-injected mice. Scale bar = 200 µm. **e** RNA expression of GFRAL, RET, and Runx2 in MCOs cultured in normal (OB-1) and osteoblast differentiation medium (OB-2) using β-actin as a control. **f** MCOs were transfected with siRNA targeting GFRAL and control siRNA; after 48 h of transfection, the cell lysates were analyzed for GFRAL, pERK, pAKT, and the respective total forms. β-Actin was used as a loading control. **g** mRNA expression analyses of *ALP, CCL2, and RANKL* in MCOs after 48 h of GFRAL siRNA transfection. The data represent the mean ± SEM. *P* ≤ 0.05 was considered to indicate statistical significance

Osteoblasts secrete RANK/receptor activator of NF-kappaB ligand (RANKL) and osteoprotegerin (OPG), which are known to influence osteoclast formation and function^6^. A healthy skeleton depends on a balanced ratio of RANKL and OPG. Treatment with rhGDF15 resulted in the upregulation of RANKL in MCOs, but no effect was observed on OPG expression (Fig. 6a). We next determined whether GDF15 contributes to the PCa-mediated upregulation of RANKL expression in osteoblasts. Treatment of MCOs with CM from PCa cells (C4-2B and LNCaP C-81) led to increased osteoblastic RANKL expression. However, treatment with CM from GDF15-KO PCa cells significantly decreased RANKL expression in MCOs compared to that achieved with parental PCa cell lines, and exogenous supplementation with rhGDF15 rescued RANKL expression in MCOs (Figs. 6b and S6a). Although CM from parental PCa cells (C4-2B and LNCaP C-81) did not alter osteoblastic OPG expression, it did increase the RANKL/OPG ratio compared to that achieved with CM from GDF15-KO PCa cells in the bone microenvironment, reflecting increased resorption and decreased bone mass (Fig. 6b and S6a).

Furthermore, we examined the expression of RANKL and OPG in the tibiae of C4-2B- and C4-2B (GDF15 KO)-injected mice. Interestingly, RANKL expression was higher in the bone of C4-2B PCa-injected mice than in that of PBS-injected or C4-2B (GDF15 KO)-injected mice (Fig. S6b). Similar to the in vitro observations with MCOs, the expression of OPG was not significantly altered in tibiae injected with C4-2B (GDF15 KO) vs. control tibiae; however, the RANKL/OPG ratio was lower in the tibiae injected with GDF15 KO cells than in those injected with parental cells (Fig. S6b). These results suggest that GDF15 from PCa cells mediates osteoclast formation and bone resorption by modulating the RANKL/OPG system. Furthermore, high alkaline phosphatase levels and osteocalcin-positive cells were observed in the tibiae from C4-2B-injected mice compared to the tibiae from C4-2B GDF15 KO-injected mice, suggesting that the increase in RANKL expression is due to GDF15-mediated osteoblast differentiation. (Fig. 6c)

Recent reports have indicated that GFRAL and RET act as a receptor and coreceptor of GDF15, respectively^18–21^; however, the presence and role of these receptors in bone cells have not yet been reported. IHC analysis showed that GFRAL and RET were present on the tibial sections of PC3-injected mice (Fig. 6d). Furthermore, we validated GFRAL staining by in situ hybridization in the isolated tibiae of mice using an RNA scope probe (Fig. S6c). MCOs expressed both GFRAL and RET, along with Runx2 (Fig. 6e). To understand the mechanism of downstream signaling of GDF15/GFRAL, we knocked down GFRAL in MCOs and observed that silencing GFRAL decreased the phosphorylation of AKT^S473^ and ERK^T202/Y204^ (Fig. 6f). Furthermore, inhibition of GFRAL suppressed the rhGDF15-mediated enhancement of *ALP, RANKL*, and *CCL2* mRNA expression (Fig. 6g). Together, these results suggest that the GDF15 receptor is present on osteoblastic precursors and might regulate the observed effect of GDF15 on bone cells.

## Discussion

PCa patients frequently develop bone metastasis, which is a major cause of morbidity and mortality. The fate of disseminated PCa is mostly determined by the interaction of tumor cells and the microenvironment of the metastatic niche. Unfortunately, the biology of PCa bone metastasis remains poorly defined. Therefore, it is critical to understand the mechanism of PCa-mediated bone metastasis and identify PCa-secreted mediators for therapeutic target identification and effective treatment development. Previously, we showed that GDF15 is highly expressed in PCa cell lines, especially in the androgen-independent LNCaP-C81 cell line and its metastatic variant LNCaP-Ln3, compared to the androgen-sensitive LNCaP-C33 cell line^15^. In contrast, GDF15 levels are low or undetectable in androgen-independent PC3 and DU145 PCa cells^15^. In accordance with published studies, we observed higher GDF15 expression in prostatic adenocarcinomas than in normal prostate tissues, and the expression increased with disease stage^31^. Similarly, high GDF15 was observed in bone metastasis specimens from PCa patients, and downregulation of GDF15 has been shown to improve the cytotoxic effect of docetaxel^31,32^. These findings raise the possibility that PCa-secreted GDF15 can regulate bone remodeling in metastatic bone lesions.

A recent report showed that PCa induced the secretion of GDF15 from terminally differentiated osteocytes, which stimulated PCa growth and invasion by stimulating early growth response 1 (EGR1) expression in PCa cells and subsequently driving the vicious cycle of bone metastasis^33^. However, the effects of PCa-secreted GDF15 in the bone microenvironment were not examined. Our study demonstrates the role of PCa-secreted GDF15 in bone metastasis and modulation of bone cell function and the microenvironment.

Our findings demonstrate that overexpression of GDF15 augments PCa metastasis to bone. It is well known that PC3 cells increase their osteolytic activity when metastasizing to bone^34^. We observed that overexpression of GDF15 increases PCa-mediated osteoclast formation and activity, as indicated by high *TRAP* and *cathepsin K* staining in bone. PCa patients with high serum levels of P1NP and CTX have significantly lower survival rates than patients with normal levels of these bone biomarkers^35^. We found increased serum levels of PINP and CTX with overexpression of GDF15 in PCa cells, which predicted the appearance of skeletal-related events, as seen in PCa patients with bone metastasis.

We observed that the GDF15-null PCa cell lines (C4-2B and LNCaP-C81) exhibited a profoundly reduced ability to grow in the bone microenvironment. Although PCa characteristically forms osteoblastic lesions when it metastasizes to bone, the PCa-secreted factors that regulate osteoclastogenesis remain unclear. C4-2B cells have been reported to induce a mix of osteoblastic and osteolytic lesions within the bone microenvironment^36^. Another report has shown that tibiae injected with C4-2B cells do not show a change in BMD compared with that of normal tibiae^37^. In line with other previous studies, we observed that bone from C4-2B-injected mice exhibited a decreased BMD and trabecular volume^38,39^. We analyzed all bone parameters through µCT and found that GDF15 is necessary for the PCa-mediated deterioration of trabecular bone. It is well established that bone resorption is a prerequisite for establishing PCa in the bone microenvironment, although the mechanism and relationship to the osteoblastic component remain unclear. Once cancer cells metastasize to bone, the interaction and cross-communication between PCa cells and the bone microenvironment result in a ‘vicious cycle,’ which leads to bone destruction and the growth and survival of cancer cells^40^. Cathepsin K-deficient mice exhibit defective bone resorption, which establishes cathepsin K as a marker of functional osteoclasts^41,42^. Our studies demonstrate that PCa-GDF15 modulates cathepsin K expression in the bone microenvironment. These in vivo findings strongly signify the role of GDF15 in PCa-induced osteoclast formation, which provides a niche for the growth of PCa.

Chemokines recruit macrophages and monocytes (precursors of osteoclasts), influencing them toward active bone remodeling for bone matrix resorption. We found that GDF15 increases the levels of C-C family chemokines, including monocyte chemoattractant protein-1 (MCP-1/CCL2), a potent chemotactic factor for monocytes in the bone microenvironment^43^. Osteoblastic CCL2 mediates both the catabolic and anabolic action of parathyroid hormones^43,44^. Several lines of evidence suggest that autocrine and paracrine functions of CCL2 are required for PCa growth and invasion, and inhibition of CCR2 reduces CCL2-mediated PCa growth and invasion^45,46^. Multiple studies have suggested that CCL2 is responsible for macrophage recruitment and PCa growth in the bone microenvironment^26–29^. CCL2 promotes macrophage fusion, as demonstrated by the reduced ability of recruited macrophages to form foreign body giant cells in CCL2-null mice^30^. Recently, it has been shown that a distinct population of bone-resident F4/80^+^ macrophages, termed osteomacs, reside on the endosteal surface and modulate osteoblast activity in normal bone remodeling as well as following injury or damage to the bone^47^. These osteomacs build canopy-like structures around osteoblasts engaged in active remodeling^47,48^. Osteomacs are distinct from osteoclasts and express common macrophage markers such as F4/80 and CD68, but not TRAP^49^. We found that compared to GDF15-null PCa cells, PCa cells expressing GDF15 increased the numbers of F4/80^+^ macrophages within mouse tibiae. CD68 is a member of the lysosome-associated membrane protein family and is required for the normal morphology and function of osteoclasts, and CD68^−/−^ mice show impaired osteoclast activity (bone resorption) and increased bone mass^50^. Mouse tibiae injected with GDF15-deleted PCa cells showed fewer CD68^+^ macrophages in the bone microenvironment. These results suggest that the deletion of GDF15 impaired the ability of PCa to recruit osteomacs and stimulate osteoclast formation. Our findings demonstrate that GDF15 is essential for PCa cells to recruit monocytes/macrophages and that it promotes osteoclast function in bone, probably via the upregulation of CCL2.

GDF15 has been shown to promote or inhibit osteoclast or osteoblast differentiation^51^, depending on the study^52,53^. Wakchoure et al. found that GDF15 induces osteoclast activation and osteoblast differentiation in bone marrow cells isolated from the long bones of 10–12-week-old C57BL/6 J mice^52^. Another study has reported that GDF15 promotes osteoclast differentiation and inhibits osteoblast differentiation of peripheral blood mononuclear cells and human bone marrow-derived mesenchymal stem cells^51^. This observed discrepancy might be due to the use of different cells for osteoblast differentiation. The role of GDF15 in these bone cells is not very clear. Thus, we isolated preosteoblasts from neonatal mouse calvaria and found that GDF15 increased the differentiation and mineralization of osteoblasts. In addition, GDF15 promoted the osteogenic differentiation of bone marrow stromal cells in a heterogeneous culture that contained monocytes and macrophages. These results suggest that PCa-secreted GDF15 has osteogenic activity, which facilitates cancer cell proliferation in the bone microenvironment.

In the bone microenvironment, osteoblasts control the development of osteoclasts from macrophages and preosteoclasts by releasing RANKL and the decoy receptor OPG. RANKL binds to its receptor RANK present on macrophages and preosteoclasts and is necessary for osteoclast formation. Altered expression of RANKL and OPG causes an imbalance between bone formation and resorption, leading to osteolytic bone metastasis in PCa^54^. We found for the first time that GDF15 significantly increased the RANKL/OPG ratio (an indicator of bone resorption) in osteoblasts. These results demonstrate an additional indirect role of GDF15 in osteoclastogenesis via the RANKL/OPG axis. GDF15 also contributed to the loss of bone microarchitecture, specifically trabecular bone, and enhanced bone resorption induced by PCa. Notably, it has been reported recently that glial-derived neurotrophic factor receptor alpha-like (GFRAL) acts as a receptor of GDF15^18–21^. Before GFRAL was identified as a receptor of GDF15, TGF-β receptor types I and II and intracellular SMAD signal transduction protein complexes were thought to mediate the cellular responses of GDF15^55–57^. In addition, GDF15 promotes actin cytoskeleton rearrangement through activation of FAK-RhoA-GEF-mediated signaling; however, the molecular mechanism is unclear^23^. GFRAL, upon binding with GDF15, interacts with the RET receptor to initiate downstream signaling, such as phosphorylation of ERK and AKT. However, the presence and role of the GDF15/GFRAL/RET axis in PCa progression and metastasis have not yet been reported.

GDF15 secreted from osteocytes promotes PCa growth and invasion, and the presence of GFRAL on PCa cell lines has been reported^33^. In the present study, we demonstrated the expression of GFRAL and RET receptors on bone. The ERK and AKT pathways are highly expressed in osteoblasts and promote their differentiation^58–62^. GFRAL silencing suggests that GDF15/GFRAL signaling can regulate the differentiation of MCOs by activating the ERK and AKT signaling pathways. Furthermore, silencing GFRAL reduces the GDF15-mediated upregulation of osteoblastic RANKL and CCL2 expression. It is likely that GDF15 binds to and activates GFRAL and RET receptors present on bone cells. However, many questions remain about the role and function of GFRAL and RET receptor signaling in the bone microenvironment, which needs further investigation. In light of these observations, it will be of interest to investigate GFRAL and RET receptor signaling in the context of the cancer-induced bone microenvironment and microarchitectural remodeling, and targeting the GDF15/GFRAL/RET axis could potentially provide a therapeutic avenue for reducing PCa-mediated bone metastasis.

Elevated serum GDF15 has been correlated with increased bone metastasis^17^. It will be interesting to examine whether circulating GDF15 and GDF15 produced locally in the bone microenvironment by tumor or stromal cells play similar or distinct roles in promoting PCa bone metastasis. While it will be of interest to utilize genetically engineered models with GDF15 or the receptors knock out in tumors or stromal compartments, currently available prostate cancer models do not spontaneously metastasize to bone at high frequency. Although TGF-β plays a paradoxical role in tumor development, recent studies have suggested that TGF-β signaling is essential for the development of bone metastasis, and TGF-β-directed therapies inhibit prostate cancer bone metastases^63–65^. In contrast, GDF15 is a divergent member of the TGF-β superfamily with low sequence conservation with other members of the TGF superfamily^66^, and its signaling through a recently identified novel receptor suggests its unique role in PCa bone metastasis.

**Fig. 7:**
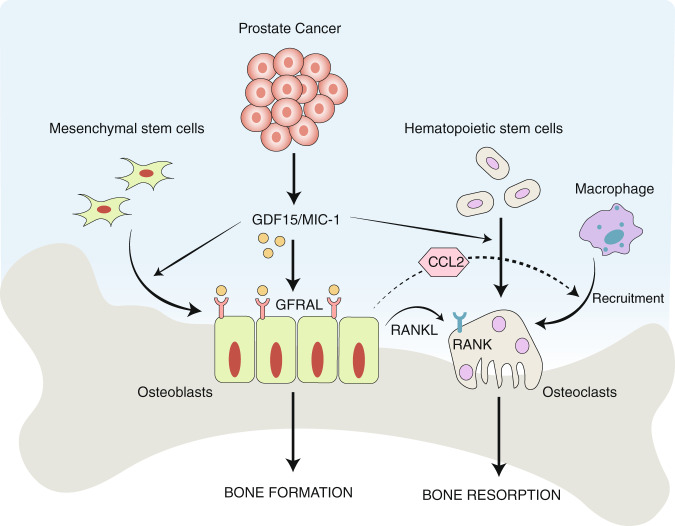
Overall mode of action of PCa-secreted GDF15 on bone metastasis. GDF15 secreted from PCa modulates the activity of bone marrow stromal cells through GFRAL/RET signaling. The GDF15-mediated increase in osteoblastic CCL2 production recruits osteomacs (bone macrophages) toward bone and causes osteolysis of bone to create a metastatic niche for PCa cells in the bone microenvironment. In addition, GDF15 also primes osteoblasts to produce high levels of RANKL that favor the growth of PCa cells in bone

In conclusion, our findings suggest that PCa cells secrete GDF15, which modulates bone marrow stromal cells (primarily osteoblasts). Osteoblasts, in turn, secrete RANKL and CCL2 to promote osteoclastogenesis and bone deterioration, favoring the growth of metastatic PCa tumor cells in the bone microenvironment (Fig. 7). Overall, these data, combined with our previously published findings, suggest that GDF15 plays a role in PCa bone metastases through bone microenvironment modulation. It will be of interest to determine the potential of targeting the GDF15/GFRAL/RET axis as a therapeutic strategy to prevent bone metastasis and increase the quality of life of PCa patients.

## Materials and methods

### Animal use and ethics

All in vivo experiments were performed as per the University of Nebraska Medical Center (UNMC) guidelines. Immunocompromised male athymic nude mice were generated and maintained under a breeding and experimental protocol approved by the UNMC Institutional Animal Care and Use Committee (IACUC), as described previously^67^.

### Cell line maintenance and preparation of conditioned media from PCa cells

Human PCa cell lines (RWPE1, DU145, and PC3) were originally obtained from the ATCC (Rockville, MD, USA), maintained in a CO_2_ incubator at 37 °C and routinely tested for STR profiles and mycoplasma contamination. The culture conditions of RWPE1, LNCaP C-81, and C42B PCa cells were as described in our earlier publications^68,69^. For conditioned medium (CM) preparation, LNCaP C-81, C42B, and PC3 cells (2 × 10^6^) were grown overnight in 100 mm culture dishes in cell culture medium. After two washes with phosphate-buffered saline (PBS), the cells were incubated for 48 h in 1% FBS in RPMI medium before collection of the CM.

### GDF15 inducible system

GDF15 was overexpressed in the PC3 cell line by using a 3rd generation lentiviral system. We performed dual transduction using rtTA along with a pLV[Tet]-mCherry:T2A:Puro>TRE3G > FLAG/hGDF15/HA:T2A:Luciferase or pLV[Tet]-mCherry:T2A:Puro>TRE3G- > Luciferase vector obtained from VectorBuilder, TX, both of which also expressed mCherry and luciferase (for quantitation and in vivo imaging). For lentiviral transduction, PC3 cells (1 × 10^5^) were seeded in six-well plates. The lentiviral mixture (1 mL) and 8 μg·mL^−1^ polybrene were added to each well. After overnight incubation, the transduction medium was replaced with fresh RPMI medium. Transduction efficiency was assessed by visualizing the cells for mCherry fluorescence, and the positive cells were sorted with a FACS Aria II (BD Biosciences). They were subsequently transduced with rtTA-containing lentivirus to activate TRE3G via the transactivator Tet3G.

### Intracardiac injections of PCa

Intracardiac injections were performed to assess skeletal metastasis, as previously described^70^. Briefly, PC-3-Luc(i) or PC3 GDF15(i) cells (1 × 10^5^) were injected into the left ventricles of male athymic nude mice (4–6 weeks old). After the intracardiac injections, the mice were kept on special water (doxycycline, 3.2 g·L^−1^, and sucrose 30 g·L^−1^) to induce GDF15 overexpression. The doxycycline-sucrose solution was changed every 3 to 4 days throughout the experiment. Skeletal metastases were monitored by bioluminescence detection of luciferase-expressing cells with the IVIS system. Bones from all major skeletal sites were harvested within 10 min after luciferin injection (150 mg·kg^−1^, i.p. injection) and analyzed for bone metastasis via bioluminescence imaging. The tibiae and femora were fixed in 10% formalin for immunohistochemistry (IHC) and then decalcified in 10% EDTA. Serum was obtained from the collected blood after cardiac puncture at the time of sacrifice to analyze the bone-related markers.

### Intratibial injections of PCa

To study the effect of GDF15 deletion on tumor growth, athymic nude male mice (4–6 weeks) were inoculated intratibially with C4-2B and C4-2B (GDF15 KO) cells. Briefly, mice were anesthetized with ketamine and xylazine, and both legs were cleaned with 70% ethanol. Luciferase-labeled PCa cell (C4-2B and C4-2B [GDF15 KO]) suspensions (5 × 10^6^ cells in 20 µL) were injected into the proximal part of the tibia using a syringe fitted with a 27 3/8-inch gauge needle. The other tibia was injected with an equal amount of PBS. Bioluminescence imaging and radiography were used to assess the tumor burden in the bone. All animals were sacrificed, and blood and bone were collected for analyses of bone markers and histology.

### Micro-computed tomography (μCT) analysis of mouse tibiae

After intratibial injection, μCT analysis of excised tibiae was performed using a Sky Scan 1172 µCT scanner (Sky Scan, Ltd., Belgium). The tibiae were dissected from the mice after euthanasia and cleaned of soft tissue. The samples were scanned at a nominal resolution (pixels) of 9.7 µm. NRECON (Skyscan) was used for the reconstruction of all images. Briefly, the reconstructed data were binarized using a threshold of 79–255. All three-dimensional volumetric analyses of trabecular bone were accomplished using CTAn software (Skyscan). The bone mineral density of trabecular bone was calculated from the binary data based on a calibration curve of calcium hydroxyapatite standards. To maintain consistency in tibial injection sites, we omitted the first 50 slices from the growth plate, after which another 258 slices were selected to analyze the data. The μCT measurements followed the guidelines of Bouxsein et al.^71^. Three-dimensional analyses were used to determine the trabecular bone architecture^72^.

### Western blot analysis

Cells were lysed with radioimmunoprecipitation assay (RIPA) buffer containing protease and phosphatase inhibitors the Lysates were freeze-thawed, syringe passaged, centrifuged at 23 000 r·min^−1^ for 25 min at 4 °C, subjected to sodium dodecyl sulfate (SDS)-polyacrylamide gel electrophoresis, and immunoblotted. Primary antibodies against GDF15 (Abcam); GFRAL (Thermo Fisher Scientific); pERK, pAKT, ERK and AKT (Cell Signaling Technology); and β-Actin (Sigma–Aldrich) were used for immunoblotting. The membranes were probed with the respective secondary antibodies for an hour before the expression was captured with enhanced chemiluminescence reagent as previously described^73^.

### Culture of calvarial osteoblasts from mice

Mouse calvarial osteoblasts (MCOs) were obtained using a previously published protocol of sequential digestion^74^. Briefly, calvaria from 1- to 2-day-old C57BL/6 J mice (5/6 of both sexes) were pooled after sacrifice by decapitation. After isolation from the skull using a midline incision, the calvaria were subjected to five sequential (10–15 min) enzymatic digestions at 37 °C in a solution containing 0.1% dispase and 0.1% collagenase. The released cells after enzymatic digestion (second to fifth digestions) were collected, centrifuged, and plated in T-25 flasks in α-MEM containing 10% FBS and 1% penicillin/streptomycin (complete growth medium).

### Osteoblast differentiation

MCOs were cultured in α-MEM supplemented with 10% FBS, and after reaching 80%–90% confluency, the cells were seeded at a density of 2 × 10^3^ cells/well in 96-well plates. The cells were treated with different concentrations of rhGDF15 and CM from PCa cells for 48 h in osteoblast differentiation medium (a-MEM supplemented with 5% FBS, 10 mmol·L^−1^ β-glycerophosphate, and 50 mg·mL^−1^ ascorbic acid). Osteoblast differentiation was assessed by determination of alkaline phosphatase (ALP) activity in cells. ALP activity was measured using p-nitrophenyl phosphate as a substrate and quantitated colorimetrically at 405 nm^75^.

### Mineralization of calvarial osteoblasts

Calvarial osteoblasts cultured until they reached 80% confluence were trypsinized and plated in differentiation medium (25 000 cells/well in a 12-well plate) consisting of complete growth medium with ascorbic acid and β-glycerophosphate. The medium was changed every alternate day for up to 21 days. The treatment group contained a medium similar to that of the GDF15 group. After completion of the experiment, the cells were washed with PBS and fixed with 4% paraformaldehyde in PBS for 15 min. The cells were stained with 40 mmol·L^−1^ (pH 4.5) alizarin red-S for 30 min and then washed with water^74^. Quantification of staining was performed by cetylpyridinium chloride (CPC) extraction as previously described^74^.

### Mineralization of bone marrow stromal cells (BMSCs)

BMSCs from 4- to 6-week-old male C57BL/6 J mice were isolated and cultured according to a previously published protocol^76^. In brief, the femora and tibiae were excised aseptically, cleaned of soft tissues, and washed in culture medium. Bone marrow was flushed out in 20 mL of α-MEM. The BMSCs were counted and plated (2 × 10^6^ cells/well) in 12-well plates in culture medium consisting of α-MEM supplemented with 10% FBS, dexamethasone (10^−7^ mol·L^−1^), ascorbic acid (50 mg·mL^−1^), and β-glycerophosphate (10 mmol·L^−1^) in the presence or absence of CM from different PCa cells along with rhGDF15 for 21 days. The old medium was replaced every 48 h. After 21 days, the attached cells were fixed in 4% formaldehyde for 20 min at room temperature and rinsed once in PBS. After fixation, the plates were stained with 40 mmol·L^−1^ alizarin red-S, which stains areas rich in nascent calcium^74^.

### RNA interference (siRNA treatment)

siRNA-mediated knockdown of GFRAL was performed in MCOs. Briefly, the MCOs were transfected with either target siRNA (SMARTPool, concentration 50 nmol·L^−1^) or control siRNA using ON-TARGET PLUS siRNA reagents (Dharmacon, Illinois, USA) as per the manufacturer’s instructions. The efficacy of GFRAL knockdown was assessed by western blot analysis after 48 h of transfection. For CCL2 and CCL12 knockdown, mouse BMM cultures were transfected as described above, and the cultures were continued for another 5 days with RANKL and MCSF and with or without rhGDF15 (10 ng·mL^−1^). At the end of the experiments, RNA was isolated to determine the TRAP transcript level.

### Osteoclast differentiation from mouse bone marrow macrophages (BMMs)

Bone marrow cells were isolated from 4-week-old C57BL/6 J mice, as described previously^43^. Briefly, after epiphyses of the bone were removed from the tibia and femur, the marrow cavity was flushed entirely with α-MEM using a sterile 25-gauge needle. Bone marrow macrophages (BMMs) were cultured in α-MEM containing 10% FBS. The cell cultures were incubated with macrophage colony-stimulating factor (M-CSF) (30 ng·mL^−1^) and receptor activator of NF-kappaB ligand (RANKL) (50 ng·mL^−1^) and exposed to various treatments consisting of rhGDF15 (0–100 ng·mL^−1^) and/or 20% CM from PCa cells. GDF15 was added at the start of the culture and at every medium change (every 2 days). After 7 days, cells were collected for mRNA analysis. Osteoclast differentiation was measured by determining the number of cells that had been positively stained by tartrate-resistant acid phosphatase (TRAP). Osteoclasts were identified as TRAP-positive-stained multinuclear cells (fused cells with 3+ nuclei) using light microscopy^74^.

### TRAP staining

After osteoclast differentiation, cells were fixed with 4% paraformaldehyde in PBS for 30 min at room temperature and stained for TRAP with a Leukocyte Acid Phosphatase Kit (387-A; Sigma–Aldrich) following the manufacturer’s instructions^43^.

### RNA isolation and quantitative real-time PCR analysis

An RNA isolation kit (Qiagen) was used to isolate the total RNA from different experiments. Total RNA (1 μg) was used for the synthesis of cDNA using random hexamers and TaqMan® Reverse Transcription Reagents. SYBR® Green Master Mix (Roche) was used for quantitative real-time PCR using a Bio–Rad instrument. The relative mRNA expression of genes was calculated using a formula reported previously^73^. β-Actin was used to normalize the expression levels, and the data are presented as the fold changes compared with the levels in the control samples. The details of all primers used in this study are provided in Supplementary Table S1. After PCR amplification, 20 μL of the PCR product was electrophoresed in a 2% agarose gel for conventional PCR assessment.

### Serum analyses for CCL2, CTX, and PINP

Blood samples were collected by cardiac puncture from the mice after the in vivo experiment. Sera were collected by centrifugation of blood samples at 5 000 r·min^−1^ for 10 min. Serum C-terminal telopeptide of collagen (CTX), N-Terminal Propeptide Of Type I Procollagen (P1NP) (Immunodiagnostic Systems Inc.) and CCL2 (Invitrogen) were measured by ELISA according to the manufacturer’s instructions.

### Immunohistochemistry and histology

At the end of the in vivo experiments, the tibiae and femora were harvested, placed in fresh 10% formalin, and decalcified in 10% EDTA before paraffin embedding. Specimens were sectioned (5 μm) and stained with H&E and TRAP (to identify osteoclasts; Acid Phosphatase Leukocyte Kit, Sigma). For IHC of human GDF15, CCL2, ALP, Ki67 and cathepsin K (intratibial experiment), the sections were incubated with primary antibodies overnight at 4 °C and then with a secondary antibody for 30 min at room temperature. An ABC Staining System (Vector Laboratories) was used as described previously^77^. For CD68, F4/80, GFRAL, RET, cathepsin K (intracardiac experiment) and osteocalcin IHC, sections were incubated with primary antibodies overnight at 4 °C and then with a secondary antibody for 20 min. The sections were counterstained with hematoxylin. These IHC experiments were performed using a Histo-Mouse Max AEC Detection Kit (Invitrogen)^43^. Mouse IgG (Jackson ImmunoResearch Laboratory) was used as a negative control. A similar IHC protocol was adopted for analysis of GDF15 expression in clinical samples. A commercially available prostate tissue microarray (TMA, US Biomax, Rockville) was immunostained with an anti-GDF15 antibody. Semiquantitative scoring ranging from 0-3 was performed by multiplying the staining intensity and percentage of positively scored cells, as reported previously^67^. Quantification of IHC staining was performed with ImageJ software^43^. Details of the antibodies used for immunohistochemistry are listed in Supplementary Table S2.

### RNA in situ hybridization assay

For in situ hybridization (ISH) of GFRAL, we used a commercially available single-color mouse-specific 20-pair double-Z oligonucleotide probe (NM_205844.3, target region 2-1186) (Advanced Cell Diagnostics). Briefly, 5-μm decalcified tibia sections from mice were used for ISH. The slides were baked for 1 h at 60 °C, and for deparaffinization, the sections were treated with protease for 30 min at 40 °C. Preheated target probes (GFRAL) were hybridized for 2 h at 40 °C, and then a series of signal amplification and washing steps were performed using a HybEZ Hybridization System. The hybridization signals of the probes were detected by sequential chromogenic reactions using brown chromogens. For detection of GFRAL RNA transcripts, we used a commercially available kit (in situ hybridization with RNAscope® 2.5 HD Assay- BROWN, RNAscope®)^78^. Brightfield images were acquired using a 40x objective.

### Statistical analysis

Student’s *t* test or one-way ANOVA was applied to evaluate differences using the GraphPad InStat software program (GraphPad Software, Inc.). *P* values < 0.05 were considered to indicate statistical significance. All in vitro assays were repeated a minimum of two times. For in vivo assays, *n* indicates the number of samples from different mice.

### Study approval

All animal experiments were reviewed and approved by the Institutional Animal Care and Use Committee (IACUC) of UNMC.

## Supplementary information

GDF15 expression in metastatic PCa patients

3D reconstruction and µCT analysis of excised tibiae of C4-2B- and C4-2B (GDF15 KO)-injected mice

GDF15 increases the expression of C-C family chemokines from osteoblasts

GDF15 promotes the osteogenic differentiation of bone marrow stromal cells (BMSCs)

Efficacy of siRNA knockdown

GDF15 increases RANKL/OPG ratio in osteoblasts

The mouse-specific primer sequences of various genes used for RT-qPCR

Antibodies used in this study

C4-2B (Parental) injected tibia

C4-2B (GDF15 KO) injected tibia
