# Supplementary figures and images for: GDF15 promotes prostate cancer bone metastasis and colonization through osteoblastic CCL2 and RANKL activation
Source: Bone Res. 2022 Jan 20;10:6. doi: 10.1038/s41413-021-00178-6 (PMC8776828; doi:10.1038/s41413-021-00178-6)

# Supplementary figure 1

a

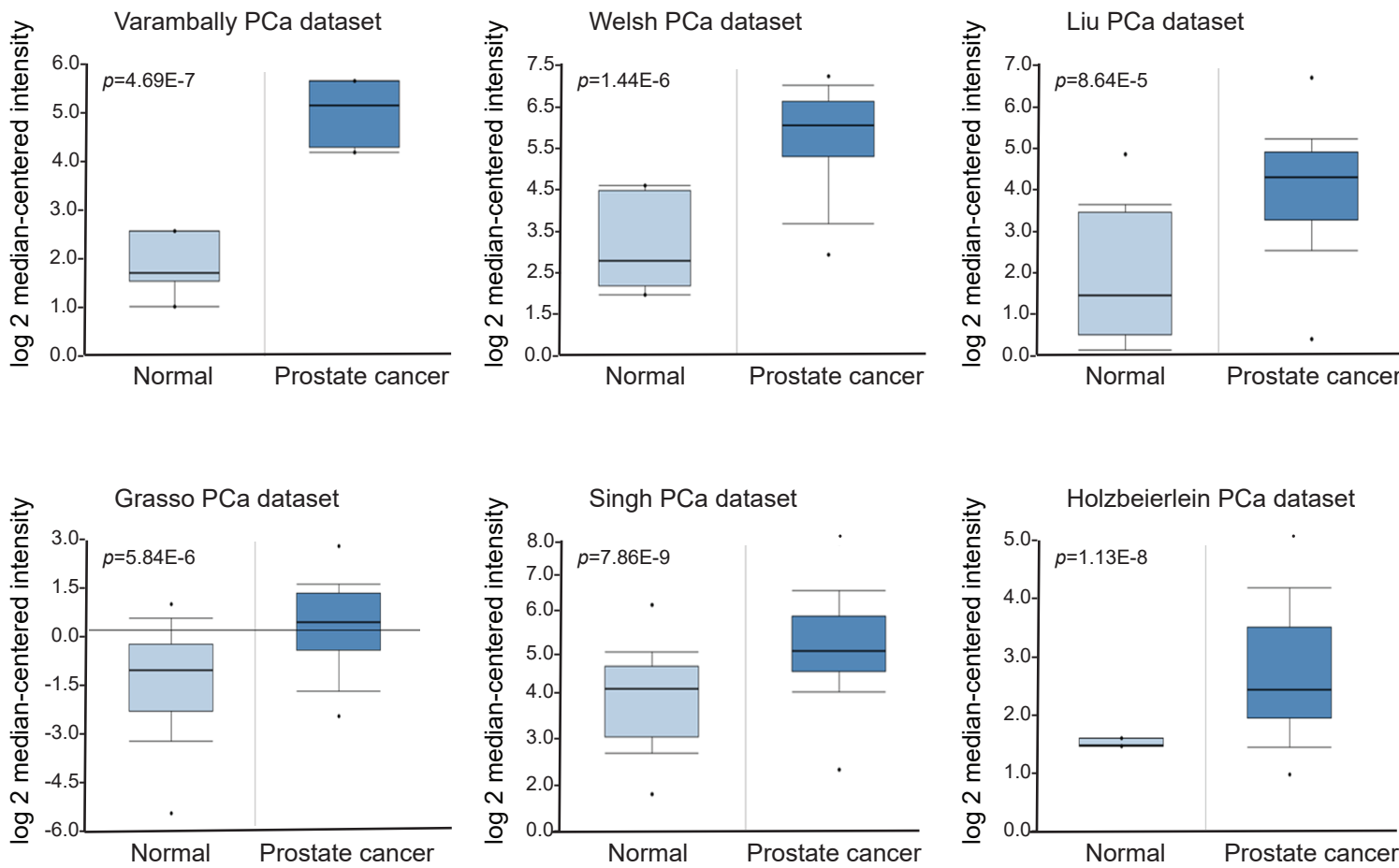

b

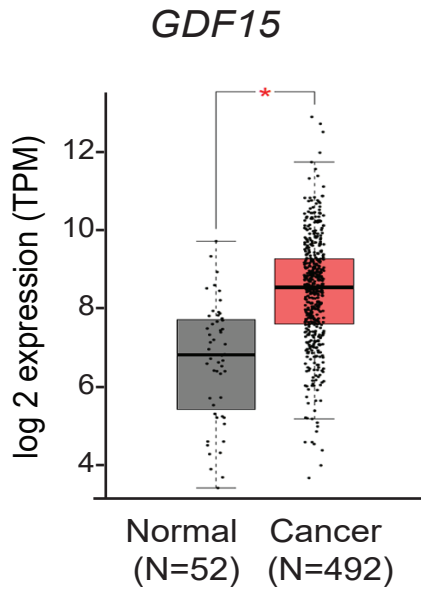

c

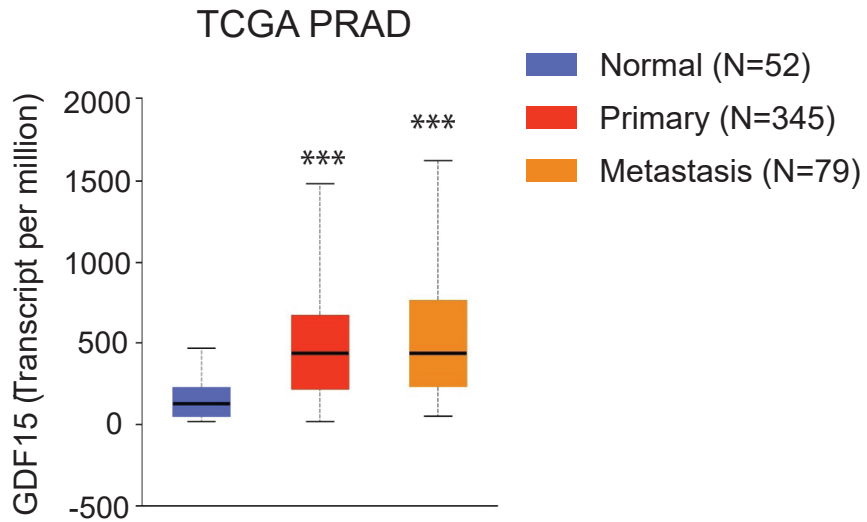

d

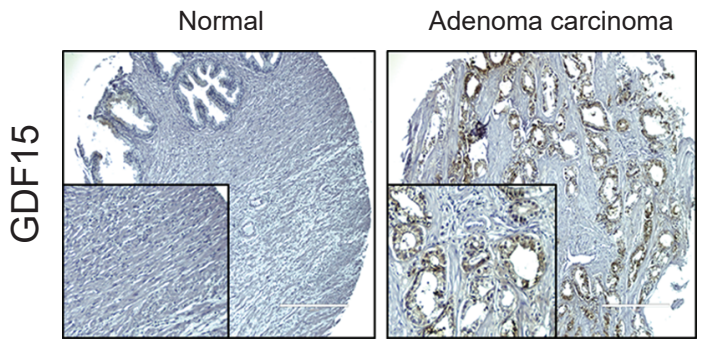

e

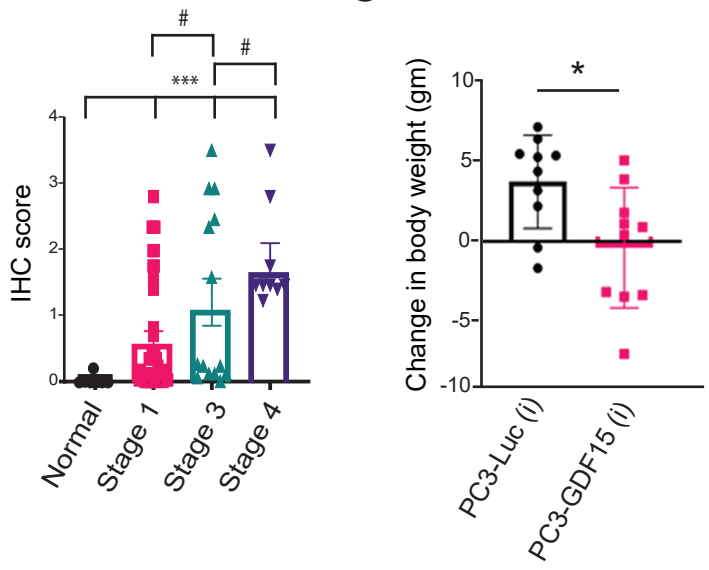

Supplement: Supplementary file 1 — GDF15 expression in metastatic PCa patients [file 41413_2021_178_MOESM1_ESM.pdf]

# Supplementary figure 2

**a**

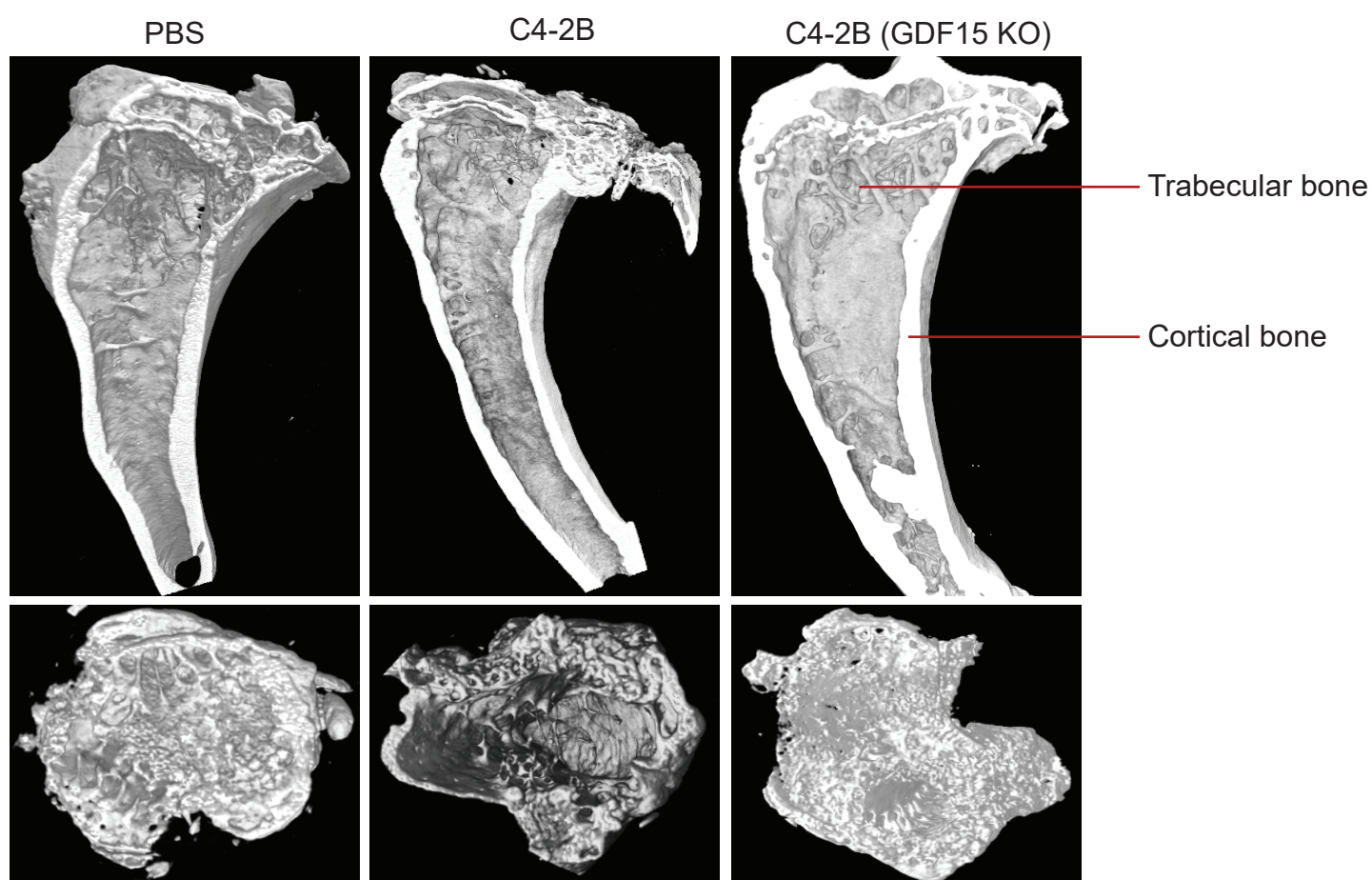

**b**

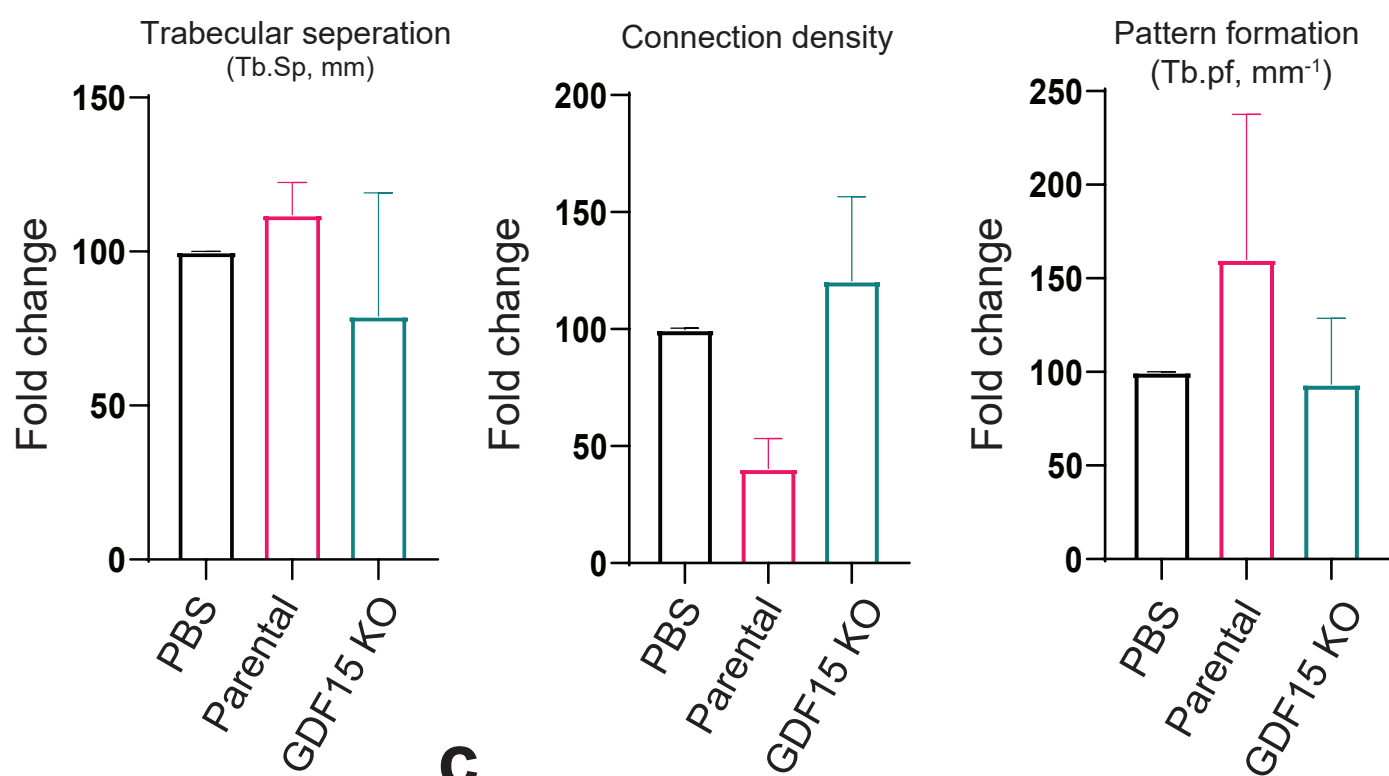

**c**

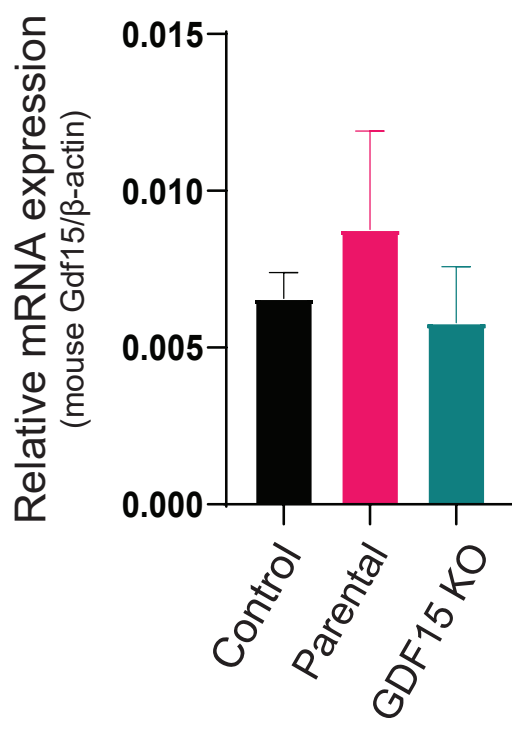

Supplement: Supplementary file 2 — 3D reconstruction and µCT analysis of excised tibiae of C4-2B- and C4-2B (GDF15 KO)-injected mice [file 41413_2021_178_MOESM2_ESM.pdf]

# Supplementary Figure 3

a

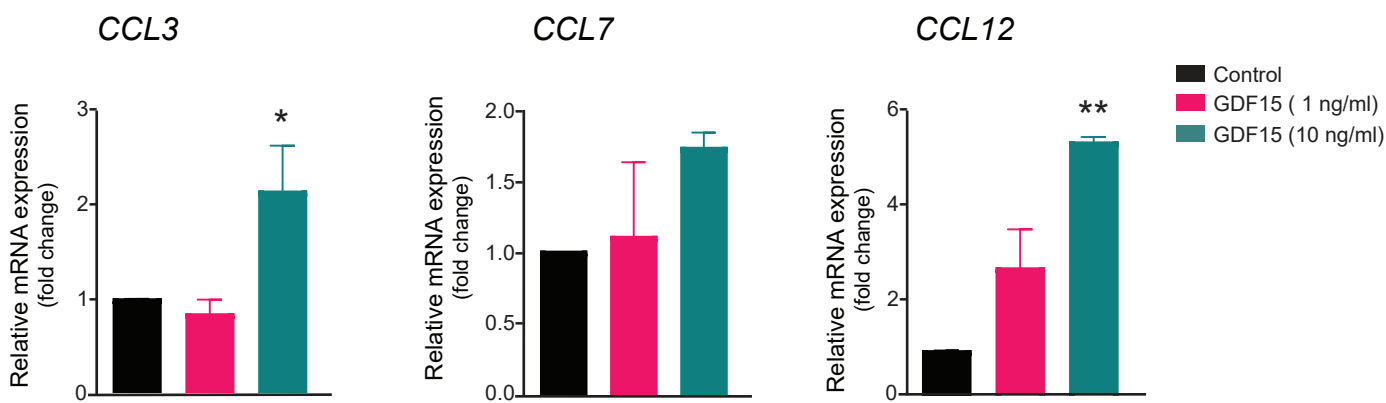

b

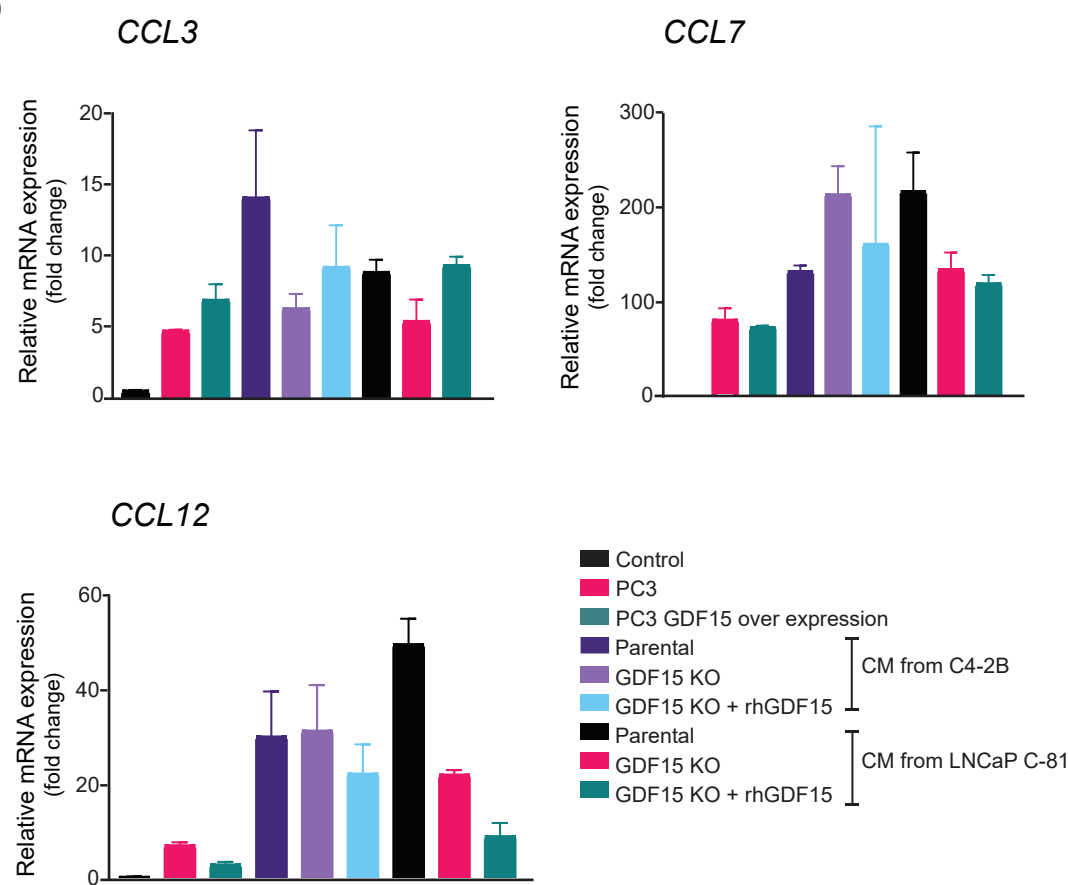

c

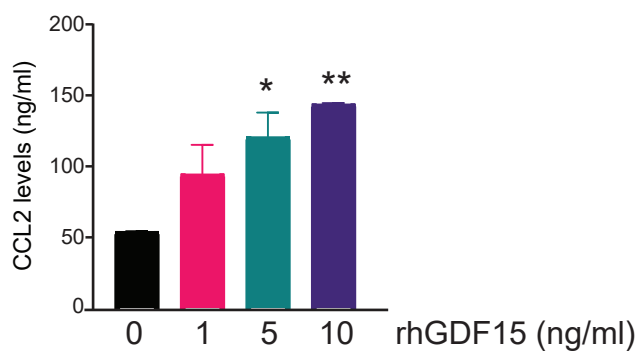

d

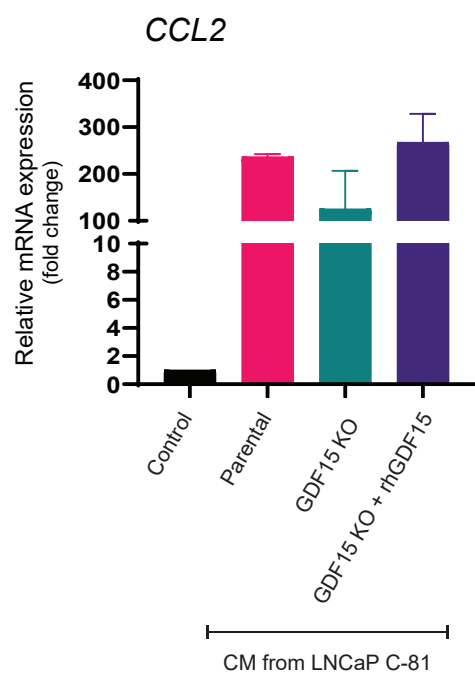

Supplement: Supplementary file 3 — GDF15 increases the expression of C-C family chemokines from osteoblasts [file 41413_2021_178_MOESM3_ESM.pdf]

# Supplementary figure 4

**a**

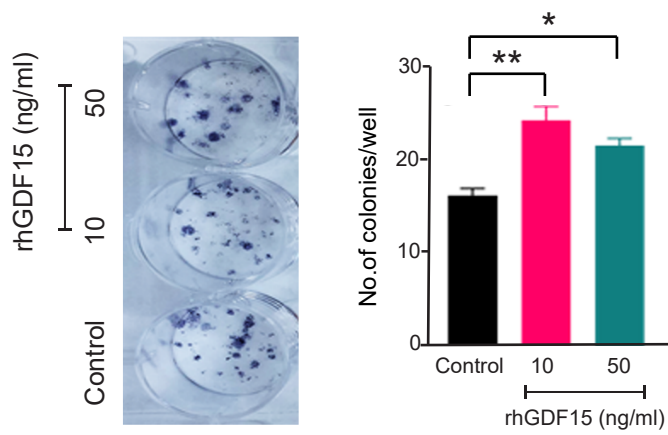

**b**

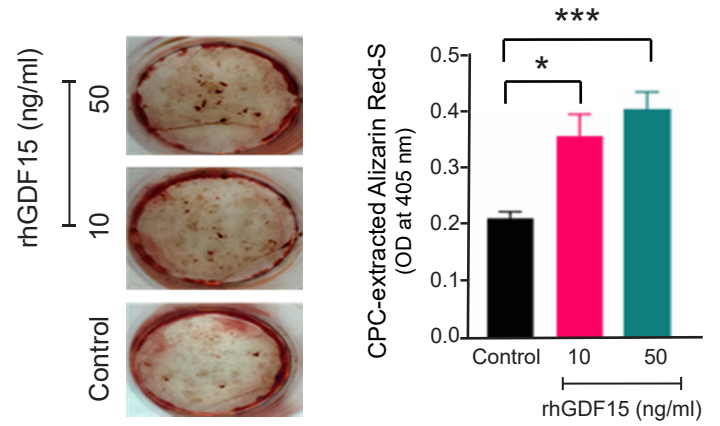

**c**

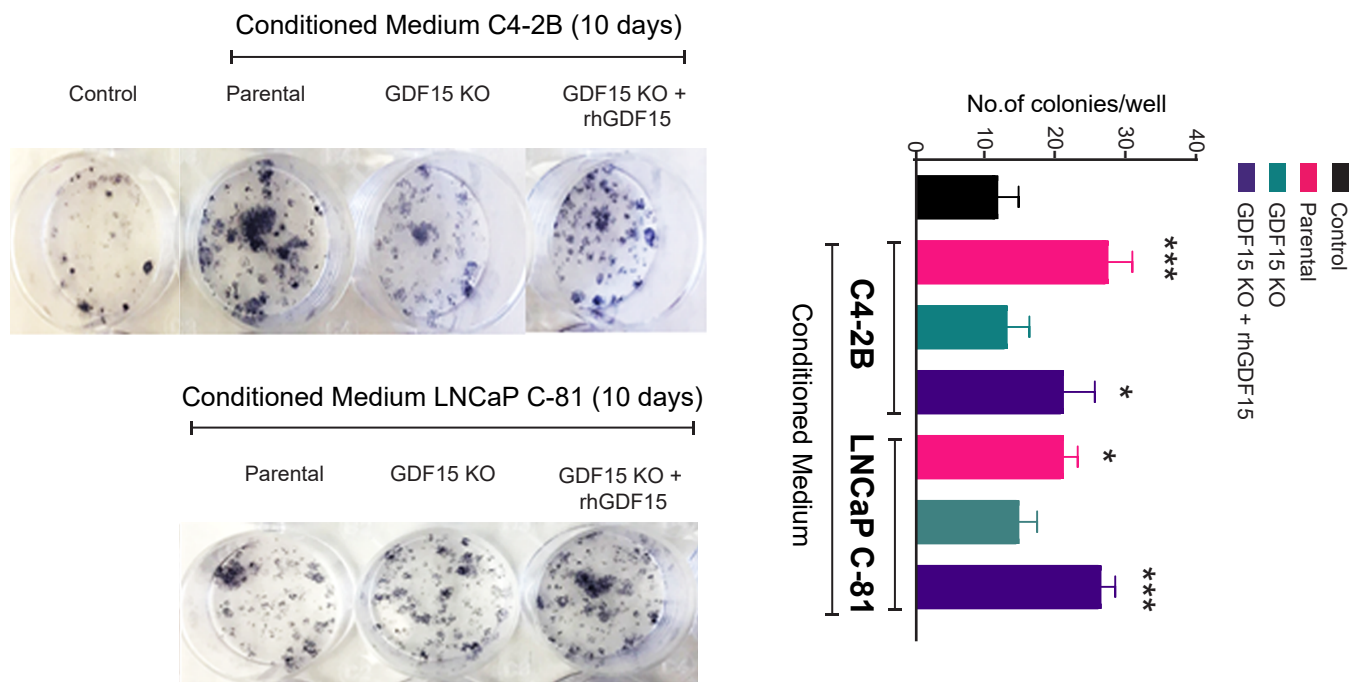

**d**

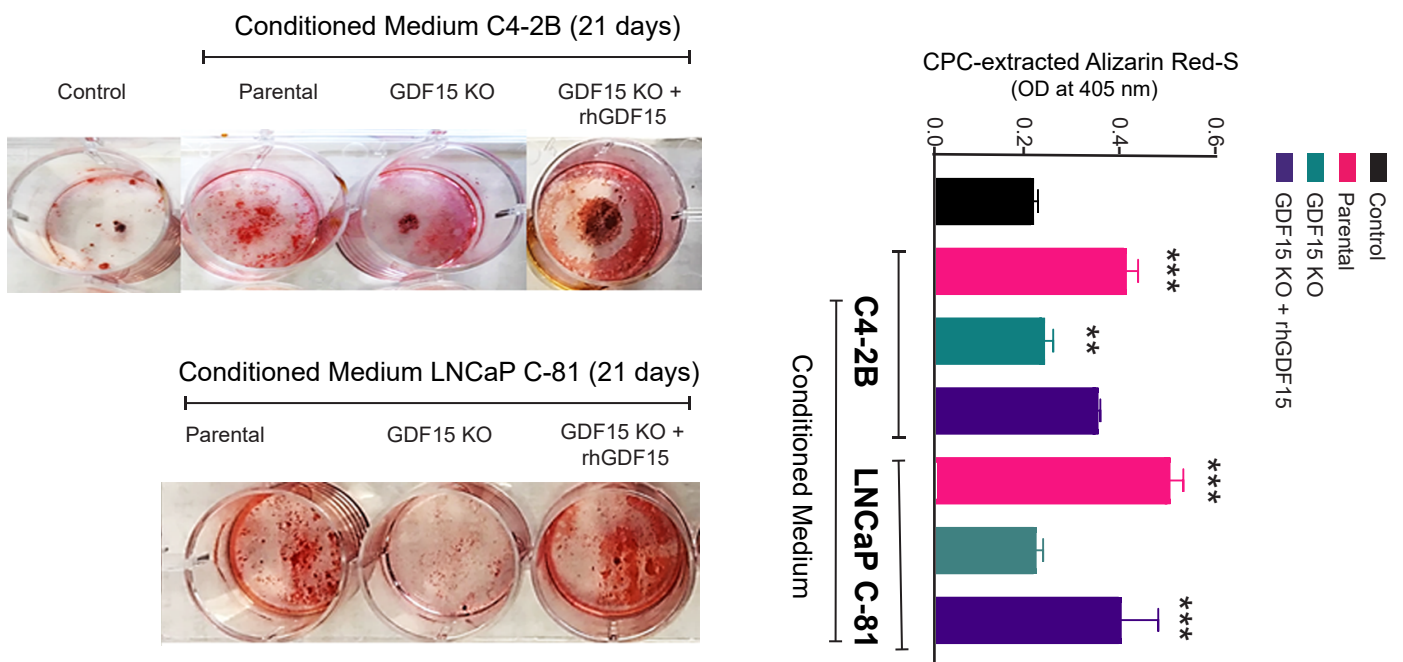

Supplement: Supplementary file 4 — GDF15 promotes the osteogenic differentiation of bone marrow stromal cells (BMSCs) [file 41413_2021_178_MOESM4_ESM.pdf]

# Supplementary figure 5

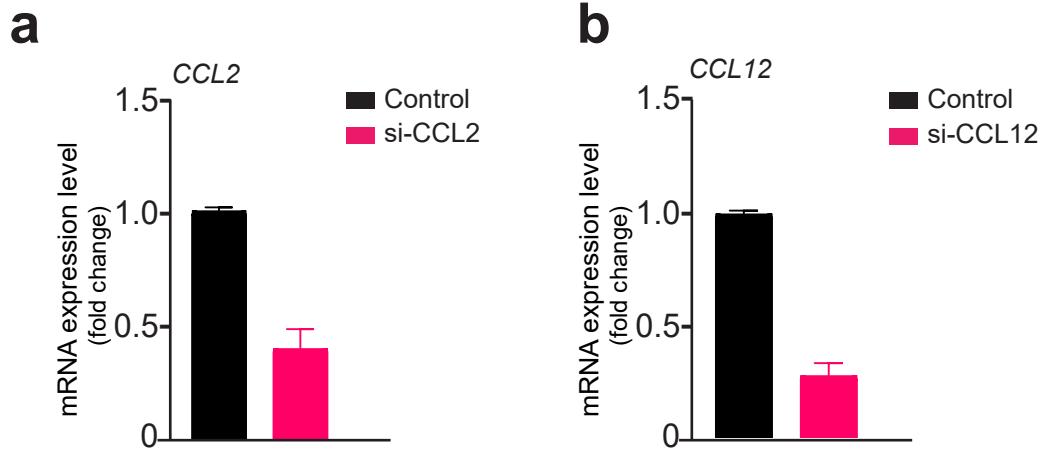

Supplement: Supplementary file 5 — Efficacy of siRNA knockdown [file 41413_2021_178_MOESM5_ESM.pdf]

# Supplementary figure 6

**a**

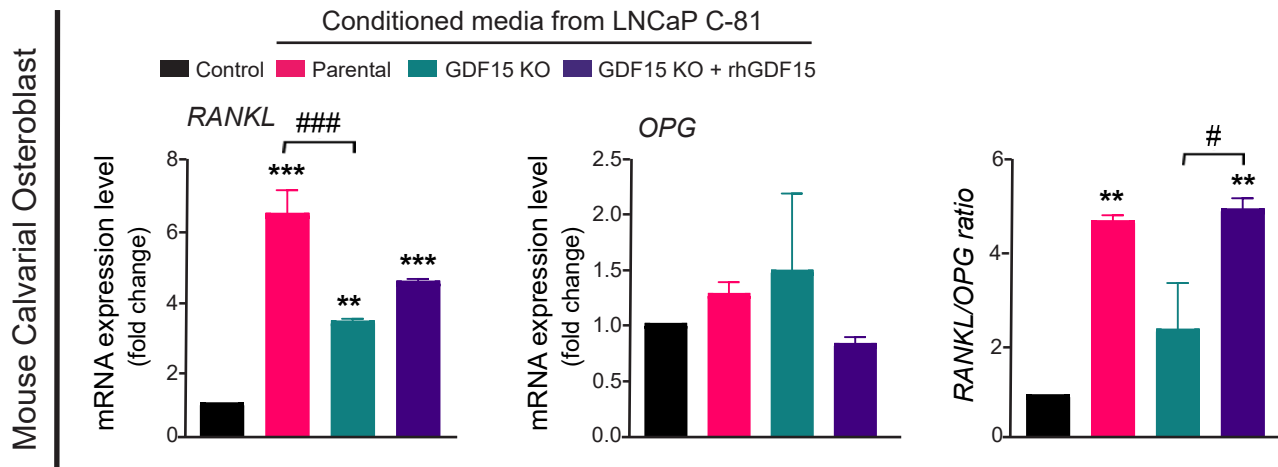

**b**

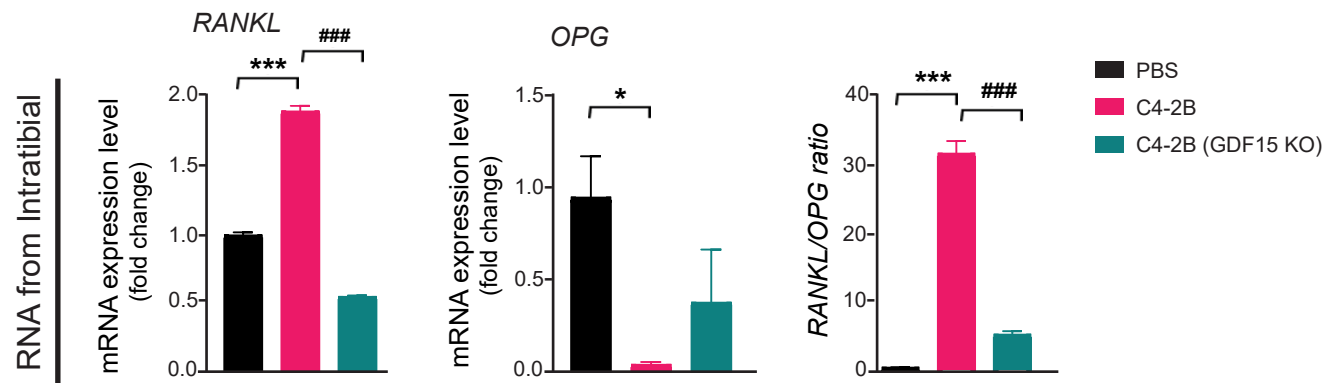

**c**

In-situ hybridization (GFRAL)

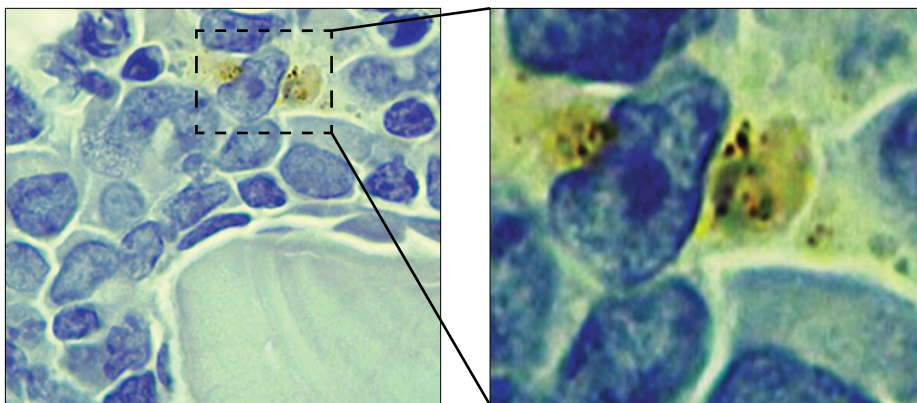

Supplement: Supplementary file 6 — GDF15 increases RANKL/OPG ratio in osteoblasts [file 41413_2021_178_MOESM6_ESM.pdf]
