# Supplementary material for: GDF15 promotes prostate cancer bone metastasis and colonization through osteoblastic CCL2 and RANKL activation
Source: Bone Res. 2022 Jan 20;10:6. doi: 10.1038/s41413-021-00178-6 (PMC8776828; doi:10.1038/s41413-021-00178-6)
Supplement: Supplementary file 7 — The mouse-specific primer sequences of various genes used for RT-qPCR [file 41413_2021_178_MOESM7_ESM.docx]

**Supplementary Table S1:** The mouse-specific primer sequences of various genes used for RT-qPCR

| **Gene name** | **Primer sequence** | **Accession number** |
| --- | --- | --- |
| *β-actin* | F 5′-TCC TCC TGA GCG CAA GTA CTC T-3′  R 5′-CGG ACT CAT CGT ACT CCT GCT T-3′ | NM_007393.5 |
| *OPG* | F 5′-GGC TGA GTG TTT TG GTG GA CAG-3  R 5′-GCT GGA AGG TTT GCT CTT GT GA-3′ | NM_008764.3 |
| *RANKL* | F 5′- TGTACTTTCGAGCGCAGATG -3′  R 5′- ACATCCAACCATGAGCCTTC-3′ | NM_011613.3 |
| *ALP* | F 5′- ATCTTTGGTCTGGCTCCCATG-3′  F 5′- TTTCCCGTTCACCGTCCAC-3′ | NM_007431.3 |
| *Osteocalcin* | F 5′- GCAATAAGGTAGTGAACAGACTCC-3′  F 5′- GTTTGTAGGCGGTCTTCAAGC-3′ | NM_001032298 |
| *Runx2* | F 5′- AGTCCCAACTTCCTGTGCTCC-3′  R 5′- CGGTAACCACAGTCCCATCTG-3′ | NM_001146038 |
| *CCL2* | F 5′- CAT CCA CGT GTT GGC TCA-3′  R 5′- GAT CAT CTT GCT GGT GAA TG AGT -3′ | NM_011333.3 |
| *CCL3* | F 5′- CCATATGGAGCTGACACCCC-3′  R 5′- GAGCAAAGGCTGCTGGTTTC-3′ | NM_011337.2 |
| *CCL7* | F 5′- CCATCAGAAGTGGGTCGAGG-3′  R 5′- ACCATTCCTTAGGCGTGACC-3′ | NM_013654.3 |
| *CCL12* | F 5′- ACACTGGTTCCTGACTCCTCT-3′  R 5′- ACCTGAGGACTGATGGTGGT-3′ | NM_011331 |
| *GFRAL* | F 5’- CTACCGAACATTCCAGACAGAA -3’  R 5’- CTCACTCCCAGAACAAGTAAGG -3’ | NM_205844.3 |
| *RET* | F 5’- AATGGTGGTTTCCCCCTGCT – 3’  R 5’- TCGTTGATGAAGGAGAAGTACACA - 3’ | NM_009050.2 |
| *TRAP* | F 5′- GGT CAG CAG CTC CCT AGA AG-3′  R 5′- GGA GTG GGA GCC ATA TGA TTT-3′ | NM_001102405.1 |
| *Cathepsin K* | F 5′- CGA AAA GAG CCT AGC GA ACA-3′  R 5′- TGG GTA GCA GCA GAA AC TTG-3′ | NM_007802.4 |
| *Nfatc1* | F 5′- TCC ACA GTC AT TTG CTC TGC-3′  R 5′- TCC AGC AGG AGG CTA TG TG-3′ | NM_016791.4 |
| *Carbonic anhydrase* | F 5′- TGGTTCACTGGAACACCAAA -3′  R 5′- AGCAAGGGTCGAAGTTAGCA-3′ | NM_001357334 |
