# Supplementary material for: GDF15 promotes prostate cancer bone metastasis and colonization through osteoblastic CCL2 and RANKL activation
Source: Bone Res. 2022 Jan 20;10:6. doi: 10.1038/s41413-021-00178-6 (PMC8776828; doi:10.1038/s41413-021-00178-6)
Supplement: Supplementary file 8 — Antibodies used in this study [file 41413_2021_178_MOESM8_ESM.docx]

**Supplementary Table S2: Antibodies used in this study**

| **Target** | **Source** | **Reactivity** | **Application** | **Vendor** | **Dilutions** |
| --- | --- | --- | --- | --- | --- |
| GDF15 | R | H, M | Immunoblot | Abcam, Massachusetts, USA | 1:1000 |
| GDF15 Human (G-5) | M | H | IHC | Santa Cruz, Texas, USA | 1:100 |
| β-actin | M | H, M | Immunoblot | Sigma-Aldrich, MO, USA | 1:5000 |
| GFRAL | R | H, M | IHC | Millipore Sigma, St. Louis, MO, USA | 1:200 |
| GFRAL | R | H, M | Immunoblot | Thermo Fischer Scientific | 1:1000 |
| RET | R | H, M | IHC | Cell Signaling Technology, Massachusetts, USA | 1:100 |
| F4/80 | M | H, M, R | IHC | Santa Cruz, Texas, USA | 1:500 |
| CD68 | M | H, M, R | IHC | Santa Cruz, Texas, USA | 1:50 |
| Cathepsin K | M | H, M, R | IHC | Santa Cruz, Texas, USA | 1:70 |
| Ki67 | M | H | IHC | Santa Cruz, Texas, USA | 1:200 |
| CCL2/MCP-1 | M | M | IHC | Santa Cruz, Texas, USA | 1:100 |
| ALP | M | H, M, R | IHC | Santa Cruz, Texas, USA | 1:100 |
| Osteocalcin | M | M | IHC | Santa Cruz, Texas, USA | 1:75 |
| pAKT | R | H, M, R | Immunoblot | Cell Signaling Technology, USA | 1:1000 |
| pERK | R | H, M, R | Immunoblot | Cell Signaling Technology, USA | 1:1000 |
| Total AKT | R | H, M, R | Immunoblot | Cell Signaling Technology, USA | 1:1000 |
| Total ERK | R | H, M, R | Immunoblot | Cell Signaling Technology, USA | 1:1000 |

H=Human; M=Mouse; R=Rat
